# Crystal structure and enzymology of *Solanum tuberosum* inositol tris/tetrakisphosphate kinase 1 (*St*ITPK1)

**DOI:** 10.1021/acs.biochem.3c00404

**Published:** 2023-12-26

**Authors:** Hayley L. Whitfield, Raquel Faba Rodriguez, Megan L. Shipton, Arthur W.H. Li, Andrew M. Riley, Barry V.L. Potter, Andrew M. Hemmings, Charles A. Brearley

**Affiliations:** 1 School of Biological Sciences, University of East Anglia, Norwich Research Park, Norwich NR4 7TJ, U.K; 2 School of Chemistry, University of East Anglia, Norwich Research Park, Norwich NR4 7TJ, U.K; 3 Medicinal Chemistry & Drug Discovery, Department of Pharmacology, University of Oxford, Mansfield Road, Oxford OX1 3QT, UK; 4 College of Food Science and Technology, Shanghai Ocean University, Shanghai 201306, China

**Keywords:** ATP-grasp kinase, fluorescence polarization, HPLC, inositol pyrophosphate, plants

## Abstract

Inositol phosphates and their pyrophosphorylated derivatives are responsive to phosphate supply and are agents of phosphate homeostasis and other aspects of physiology. It seems likely that the enzymes that interconvert these signals work against the prevailing milieu of mixed populations of competing substrates and products. The synthesis of inositol pyrophosphates is mediated in planta by two classes of ATP-grasp fold kinase: PPIP5 kinases, known as VIH, and members of the inositol tris/tetrakisphosphate kinase (ITPK) family, specifically ITPK1/2. A molecular explanation of the contribution of ITPK1/2 to inositol pyrophosphate synthesis and turnover in plants is incomplete: the absence of nucleotide in published crystal structures limits explanation of phosphotransfer reactions and little is known of the affinity of potential substrates and competitors for ITPK1. Herein, we describe a complex of ADP and *St*ITPK1 at 2.26Å resolution and use a simple fluorescence polarization approach to compare the affinity of binding of diverse inositol phosphates, inositol pyrophosphates and analogs. By simple HPLC, we reveal novel catalytic capability of ITPK1 for different inositol pyrophosphates and show Ins(3,4,5,6)P_4_ to be a potent inhibitor of the inositol pyrophosphate-synthesizing activity of ITPK1. We further describe the exquisite specificity of ITPK1 for the *myo-*isomer amongst naturally occurring inositol hexakisphosphates.

## Introduction

Since the discovery of inositol pyrophosphates (diphosphoinositol phosphates) in plants [1–2], inositol pyrophosphates have emerged as participants in diverse aspects of plant physiology extending to phosphate starvation response, hormone signaling, symbiosis and response to pathogens [3–10]. The same can also be claimed of inositol phosphates lacking anhydride bonds [3, 11–15]. Claim of contribution of inositol pyrophosphates to aspects of plant biology rests heavily on characterization of enzymes and analysis of mutants thereof. ITPKs and PPIP5Ks, variously referred to as VIH or VIP in plants and yeast, respectively, contribute to inositol pyrophosphate synthesis. Both possess the ATP-grasp fold. PPIP5Ks contain an additional histidine acid phosphatase domain [16].

The catalytic potential of plant ITPKs is particularly diverse [17–22] when compared with the kinase activity of VIH1/2, considered only to act on InsP_6_ and 5-InsP_7_, 5-PP-Ins(1,2,3,4,6)P_5_, hereafter 5-PP-InsP_5_. For plants, VIH1/2 analysis has been restricted to isolated domains [23–24]. Like many inositol phosphate kinases, IP6K [25], IP5K (IPK1) [26], ITPKs are reversible phosphotransferases [21–22, 27] as is the ATP-grasp kinase domain of PPIP5K [28]. Consideration of reversibility under prevailing physiological conditions, with usually poorly defined nucleotide status, has the consequence that the causative signaling species among ITPK and VIH substrates/products are difficult to decipher.

In vitro, *At*ITPK1 shows greater ATP-synthesis from 5-PP-InsP_5_ than 5-PP-InsP_5_ synthesis from InsP_6_ [27]. These activities are, however, a very small fraction of the Ins(3,4,5,6)P_4_ 1-kinase activity of the enzyme [21]. To date, the only enzymes capable of synthesizing InsP_8_, 1,5-InsP_8_, 1,5-[PP]_2_-Ins(2,3,4,6)P_4_, hereafter 1,5-[PP]_2_-InsP_4_, the presumed endogenous [PP]_2_-InsP_4_ in plants [3], are the VIH1/2 enzymes [23–24]. Even so, the activities of ITPK1/2 and VIH1/2 [20–21, 27] do not account for the full spectrum of inositol pyrophosphates detected in plants [21, 27, 34]. The possible enantiomers include 1-InsP_7_, 1-PP-Ins(2,3,4,5,6)P_5_, hereafter 1-PP-InsP_5_; 3-InsP_7_, 3-PP-Ins(1,2,4,5,6)P_5_, hereafter 3-PP-InsP_5_; 4-InsP_7_, 4-PP-Ins(1,2,3,5,6)P_5_, hereafter 4-PP-InsP5; and 6-InsP7, 6-PP-Ins(1,2,3,4,5)P5, hereafter 6-PP-InsP_5_. The *meso* isomers include 5-PP-InsP_5_ and 2-InsP_7_, 2-PP-Ins(1,3,4,5,6)P_5_, hereafter 2-PP-InsP_5_.

The ITPK family, including members that synthesize inositol pyrophosphates [20–21, 27] or do not [20, 22], is represented in early land plants [29] and in early aquatic vascular plants, the duckweeds in which a lipid-independent pathway of InsP_6_ synthesis that uses the favored InsP_4_ substrate of plant ITPK1 [21] was described [30–32]. Herein, we have solved a crystal structure for a potato enzyme, *St*ITPK1, in complex with ATP. We show the enzyme’s preference for InsP_4_ over InsP_6_ and PP-InsP substrates and describe a simple, yet powerful, fluorescence polarization approach that could advance study of other ATP-grasp kinases.

## Materials And Methods

Details of protein purification, enzyme assays, HPLC analysis of reaction products, ligand-binding assays and X-ray crystallography can be found in Supplementary Information.

## Results

### *St*ITPK1 displays phospho-kinase activity

The structures of compounds tested as substrates, ligands or inhibitors of *St*ITPK1 and *At*ITPK1 are shown (Figure S1). *St*ITPK1 is similar to *At*ITPK1: generating 5-PP-InsP_5_ from InsP_6_ (Figure S2A), lacking activity against Ins(1,2,3,5,6)P_5_ (Figure S2B) and showing phospho-kinase activity against the enantiomer Ins(1,2,3,4,5)P_5_, yielding a product that eluted before InsP_6_ (Figure S2C).

Multiple inositol hexakisphosphate isomers are present in soil. In addition to *myo*-InsP_6_, D-*chiro*-InsP_6_, *neo*-InsP_6_ and *scyllo*-InsP_6_ have been identified [33]. Their presence is unexplained, but because plant matter is a major input to soil we tested whether they might be substrates for *St*ITPK1. Among isomers of inositol hexakisphosphate, *St*ITPK1 phosphorylates the *myo*-isomer only (Figure S2A,D-F). The lack of activity towards other inositol hexakisphosphates described in soil makes it unlikely that this ancestral plant enzyme, present in liverworts, bryophytes and early vascular aquatic plants [29], could contribute to the presence of non-canonical inositol pyrophosphates in soil, should they be found.

### *St*ITPK1 displays stereospecific [PP]_2_-InsP_4_/ADP phosphotransferase activity

**Figure 1:**
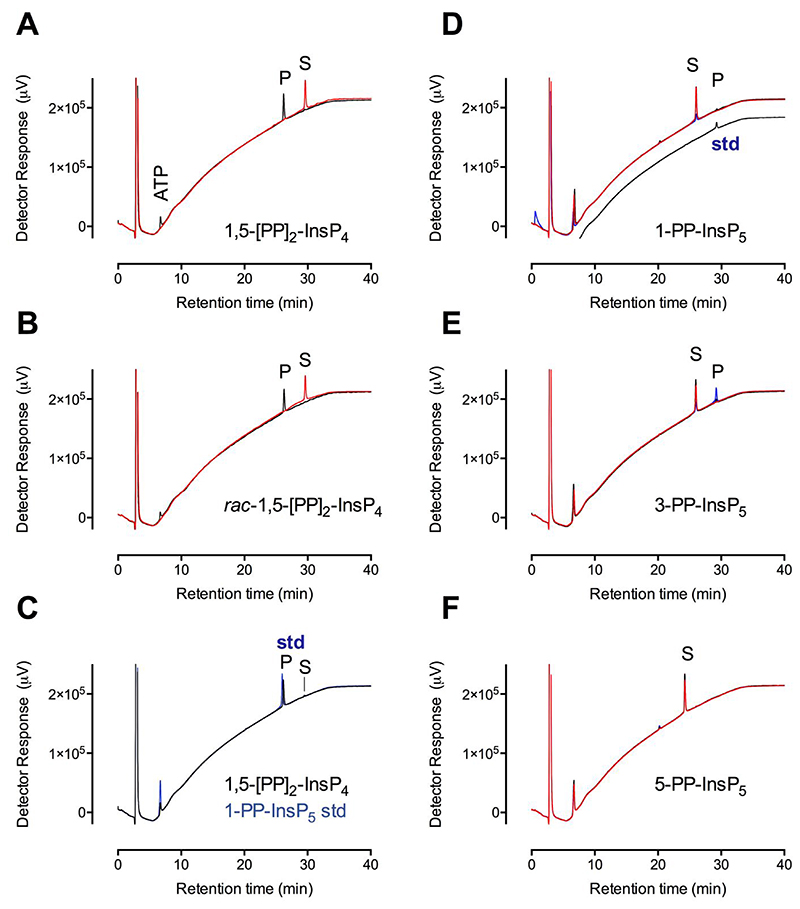
*St*ITPK1 is a reversible inositol pyrophosphate-ADP phosphotransferase. HPLC resolution of products of 12h reaction of *St*ITPK with ADP and **A**, 1,5-[PP]_2_-InsP_4_; **B**, *rac*-1,5-[PP]_2_-InsP_4_. Products of dephosphorylation of 1,5-[PP]_2_-InsP_4_ coelute with 1-PP-InsP_5_, **C**. Substrates are indicated, S; products, P; and standards, std (in blue). Chromatograms of reactions without enzyme are shown in red, with enzyme in black and chromatograms of standards are shown in blue. The position of elution of ATP formed by phosphotransfer to ADP is shown in panel A. ADP elutes in the solvent front. The standards showing elution position of PP-InsP_5_ in C contain ATP. The ATP peaks in all other panels are products of phosphotransfer from substrate to ADP. HPLC of products of reaction of *St*ITPK1 with ATP and **D**, 1-PP-InsP_5_; **E**, 3-PP-InsP_5_; **F**, 5-PP-InsP_5_. Substrates are indicated, S; products, P; and standards, std. Chromatograms of reactions without enzyme are shown in red, 3h incubations with enzyme are shown in black and 12h incubations in blue. The volume of sample injected for the 12h sample in A was approximately 30% of that for the other samples. The positions of elution of ATP and 1,5-[PP]_2_-InsP_4_ standard (trace offset on the *y*-axis) are shown in panel A. ADP elutes in the solvent front. The HPLC column was eluted with a gradient of HCl.

Recently, non-canonical PP-InsP_5_ species have been identified in plants [27, 34]. CE-MS peaks with chromatographic mobility and parent/daughter ion relationships identical with synthetic D-and or L-4-PP-InsP_5_ [4-PP-InsP_5_/6-PP-InsP_5_] and the *meso*-compound 2-PP-InsP_5_ have been detected [27, 34]. Similar conclusions can be drawn from [21]. It is, however, unknown whether plants possess a single [PP]_2_-InsP_4_, a mixture of the enantiomers 1,5-[PP]_2_-InsP_4_ / 3,5-[PP]_2_-InsP_4_ or additional isomers and enantiomeric pairs [3]. Recent work has identified a family of 5-β-phosphate-targeting phosphatases [34], showing that VIH1/2 is not the only agent of [PP]_2_-InsP_4_ turnover. Moreover, it is also unclear whether plant [PP]_2_-InsP_4_s are substrates for phosphotransfer to ADP. The known reversibility of phospho-kinase activity of *At*ITPK1 [21,27] prompted us to test the same of *St*ITPK1. We did so because the presence of 1-PP-InsP_5_ and/or 3-PP-InsP_5_ in plants [21,27] could also arise from dephosphorylation of 1,5-[PP]_2_-InsP_4_ (InsP_8_) and/or 3,5-InsP_8_, 3,5-[PP]_2_-Ins(1,2,4,6)P_4_, hereafter 3,5-[PP]_2_-InsP_4_. We tested the ability of *St*ITPK1 to effect phosphotransfer from 1,5-[PP]_2_-InsP_4_ and a racemic mixture of the two enantiomers, hereafter *rac*-1,5-[PP]_2_-InsP_4_ (Figure 1). *St*ITPK1 showed enantiospecific phosphotransfer (to ADP) activity against 1,5-[PP]_2_-P_4_, yielding 1-PP-InsP_5_ and ATP (Figure 1A,C). The same was true with *rac*-1,5-[PP]_2_-InsP_4_ (Figure 1B,C). Full conversion of *rac*-1,5-[PP]_2_-InsP_4_ (ie., of both 1,5-[PP]_2_-InsP_4_ and its enantiomer 3,5-[PP]_2_-InsP_4_) shows that both enantiomers are substrates, with enzyme attack on the 5-β-position of both. Consistent with this attack on the 5-β-phosphate, neither of the non-hydrolyzable 1,5-[PA]_2_-Ins(2,3,4,6)P_4_, hereafter [1,5-[PA]_2_-InsP_4_, nor 1,5-[PCP]_2_-Ins(2,3,4,6)P_4_, hereafter 1,5-[PCP]_2_-InsP_4_, analogs were substrates (Figure S3E,F). Both compounds eluted substantially earlier than 3-PP-InsP_5_ (or its enantiomer, 1-PP-InsP_5_) (Figure S3). Similar observations were made for *At*ITPK1 (Figure S3). Here, both chiral- and racemic 1,5-[PP]2-InsP_4_ were substrates for phospho-transfers from the *meso*-5-position, yielding 1-PP-InsP_5_ / 3-PP-InsP_5_ and ATP products. Again, the ‘non-hydrolyzable’ [PA]_2-_ and [PCP]_2_-analogs were not substrates (Figure S3C,D). These results extend the repertoire of phosphotransfer reactions catalyzed by *At*ITPK1, two groups have shown transfer of the γ-phosphate of ATP to the 5-phosphate of InsP_6_and vice versa, from the β-position of 5-PP-InsP_5_ to the β-phosphate of ADP [21, 27]. The two studies arrived at very similar values of *K*_cat_ (rate constant) for phosphorylation of InsP_6_ and the latter derived *K*_cat_ for ATP-synthesis double that for 5-PP-InsP_5_ production.

### *St*ITPK1 and *At*ITPK1 display stereospecific phospho-kinase activity against PP-InsPs

Surprisingly, given current dogma that [PP]_2_-InsP_4_ synthesis belongs only to VIH1/2, we found 1-PP-InsP_5_ and 3-PP-InsP_5_ both to be phospho-kinase substrates of *St*ITPK1 incubated with ATP (Figure 1D,E) with 1-PP-InsP_5_ the weaker substrate. Both yielded products, 1,5-[PP]_2_-InsP_4_ and 3,5-[PP]_2_-InsP_4_, respectively, that co-eluted with a 1,5-[PP]_2_-InsP_4_ standard (Figure 1D). To confirm the commonality of this activity, we tested *At*ITPK1 for the same activity. *At*ITPK1, like *St*ITPK1, also showed stronger activity against 3-PP-InsP_5_, generating a product with the chromatographic properties of 3,5-[PP]_2_-InsP_4_ (Figure S4). Neither *St*ITPK1 (Figure 1F) nor *At*ITPK1 (Figure S4C) phosphorylated 5-PP-InsP_5_. These observations establish exchange of phosphate between the γ-position of ATP and a ‘vacant β-position’ on PP-InsP_5_ species present in plants and vice versa, viz. transfer of a β-phosphate from [PP]_2_-InsP_4_ to the ‘vacant γ-position’ of ADP. That the activity resides with ITPK1 is wholly consistent with the role of ITPK1 as a master regulator of phosphate starvation response [15, 27]. The physiological balance of such reactions will be dependent on the concentrations of nucleotides and inositol pyrophosphosphates.

### A ligand-binding assay allows comparison of binding of diverse inositol phosphates and inositol pyrophosphates to ATP-grasp kinases

**Figure 2:**
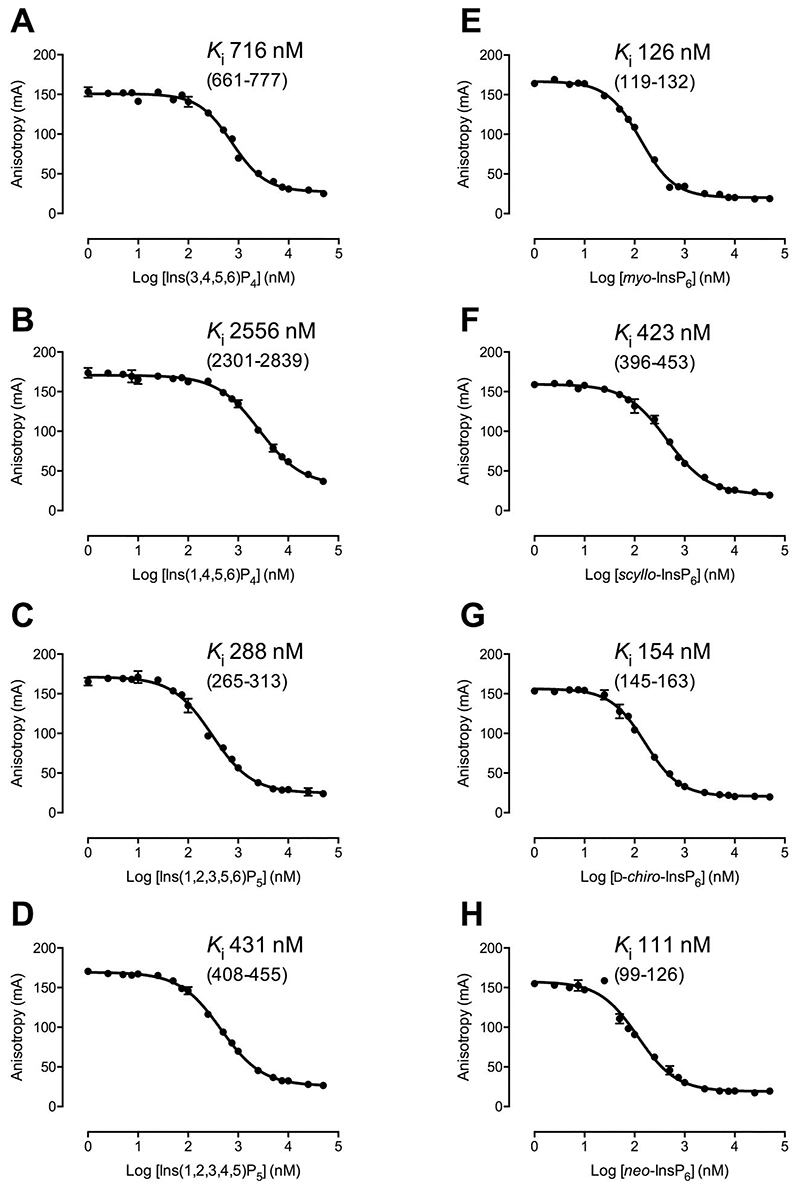
High-affinity binding of inositol phosphates to *St*ITPK1. Displacement of 2-FAM-InsP_5_ with **A**, Ins(3,4,5,6)P_4_; **B**, Ins(1,4,5,6)P_4_; **C**, Ins(1,2,3,5,6)P_5_; **D**, Ins(1,2,3,4,5)P_5_; **E**, *myo*-InsP_6_; **F**, *scyllo*-InsP_6_; **G**, D-*chiro*-InsP_6_; **H**, *neo*-InsP_6_. Data are the means and standard deviations of four replicate measurements; *K*_i_ (nM) with confidence interval (nM) in parentheses.

To determine the relative strengths of binding of different inositol phosphate and inositol pyrophosphate substrates of *St*ITPK1 we undertook fluorescence polarization experiments with 2-FAM-InsP_5_ [35]. This molecule has proved a useful probe of the active and/or inositol phosphate-binding sites of enzymes as diverse as SHIP2 [36], IP5K (*At*IPK1) [37] and HDAC complexes [35]. A saturation curve for binding of 2-FAM-InsP_5_ to *St*ITPK1 is shown (Figure S5) and displacement curves are shown for diverse inositol phosphates (Figure 2). The structures of these compounds are shown (Figure S1).

*K*_i_ ranged from 111 nM for *neo*-InsP_6_ (Figure 2H) to 2556 nM for Ins(1,4,5,6)P_4_ (Figure 2B). *K*_i_ values increased, broadly, InsP_6_ < InsP_5_ < InsP_4_. Consistent with enzymatic data for *At*ITPK1 [21], Ins(3,4,5,6)P_4_, the better substrate of the two enantiomers, bound much more tightly with *K*_i_ of 716 nM (Figure 2A) than Ins(1,4,5,6)P_4_ (Figure 2B). Interestingly, the two InsP_5_s also displayed similar binding affinity (*K*_i_ of 288 nM for Ins(1,2,3,5,6)P_5_ and 431 nM for Ins(1,2,3,4,5)P_5_) (Figure 2C,D) against enzyme activity for the latter only (Figure 1). Among inositol hexakisphosphates, with exception of the *scyllo*-stereoisomer (Figure 2F), all bound with similar affinity with *K*_i_ in the range 111-154 nM (Figure 2E-H); *scyllo*-InsP_6_ (423 nM).

We rationalize the failure of *St*ITPK1 to phosphorylate *neo*-InsP_6_ (Figure S2D), with its two axial phosphates in the plane of symmetry, equivalent to the axial 2-phosphate and equatorial 5-phosphate of *myo*-InsP_6_ (Figure S1), as being consistent with requirement for an equatorial 5-phosphate in the single *myo*-InsP_5_ substrate, Ins(1,2,3,4,5)P_5_. The failure of *St*ITPK1 to phosphorylate *scyllo*-InsP_6_ (Figure S2F), a C2-epimer of *myo*-InsP_6_ with six equatorial phosphates (Figure S1), suggests a requirement for phospho-kinase substrates to possess a single axial phosphate in the plane of symmetry (for *myo*-InsP_6_, in the 2-position). Consistent with this, D-*chiro*-InsP_6_, which has two axial phosphates in *trans*, was also not a substrate (Figure S2E).

Consistent with the displacement data and previous study of *At*ITPK1 [21], Ins(3,4,5,6)P_4_ proved a better substrate than both its enantiomer Ins(1,4,5,6)P_4_ and InsP_6_ (Figure S6). Discrimination between the InsP_4_ enantiomers was ~200-fold in favour of Ins(3,4,5,6)P_4_ for both *St*ITPK1 and *At*ITPK1 (Figure S6). This enantioselectivity is reversed for *At*ITPK4 [22]. For both *St*ITPK1 and *At*ITPK1, InsP_6_ proved to be a better phospho-kinase substrate than Ins(1,4,5,6)P_4_ is a hydroxy-kinase substrate (Figure S6).

### A nucleotide-liganded structure of *St*ITPK1 allows modeling of phosphotransfer reactions of plant ITPK1

**Figure 3:**
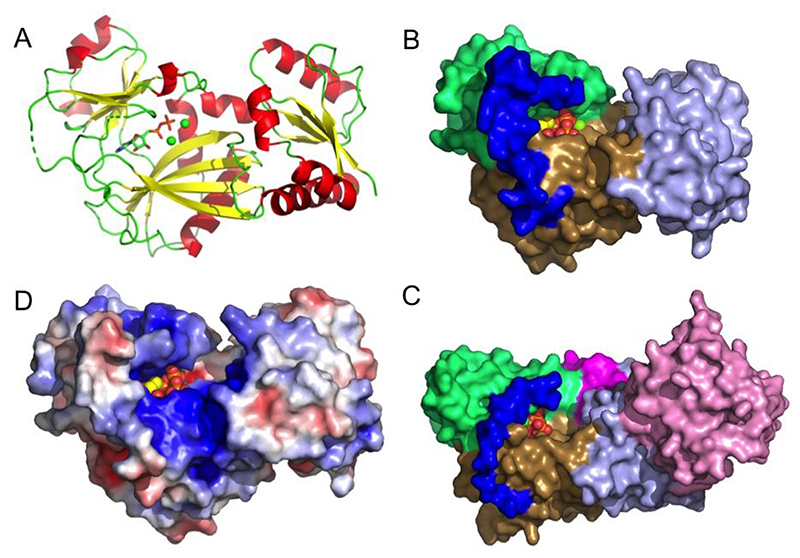
An overview of the crystal structure of *St*ITPK1. **A**, Cartoon representation of the structure of *St*ITPK1, coloured by secondary structure (α-helix red, β-sheet yellow and coil green). Broken lines in the backbone trace indicate residues unresolved in the model due to disorder. Bound nucleotide is shown in stick format with colouring as follows: carbon-green, oxygen-red, nitrogen-blue and phosphorus-orange. **B**, Molecular surface representation of the structure of *St*ITPK1 coloured by subdomain. Subdomains are: kinase N-terminal domain (light blue), kinase central domain (lime green) and kinase central domain (sand). Here and in panel D, the polypeptide connecting the central and C-terminal domains is coloured dark blue. Bound ADP is shown in atom sphere format. **C,** A molecular surface representation of the structure of *At*ITPK4 (PDB: 7PUP). Colouring as in panel C except that the additional HAD domain found in this enzyme is coloured pink and the tab insertion unique to ITPK4s is coloured magenta. **D**, The molecular surface of *St*ITPK1 coloured by electrostatic potential (red-acidic, blue-basic). The orientation of the molecule is the same as that in panel A. In panels B-D, bound ADP is shown in atom sphere format and coloured according to that in panel A.

Crystal structures have been reported for two plant ITPKs, *At*ITPK4 (PDB: 7PUP) and *Zm*ITPK1 (PDB: 7TN8) [22, 38]. The former lacks bound inositol phosphate, the latter lacks bound nucleotide. To explain the interaction of ITPK1 with nucleotide co-substrate, the *St*ITPK1 crystal structure (residues 8-320) was solved in space group C222 with a monomer of the enzyme in the asymmetric unit (Figure 3) (PDB 8OXE). Refined against all data to 2.26Å resolution, the final structural model had an R-factor of 19.0 % (Rfree 24.1 %) (Table S1). As expected, *St*ITPK1 adopts the ATP-grasp kinase fold with three conserved subdomains referred to here as N-terminal, central and C-terminal domains, following the nomenclature previously applied in descriptions of the crystal structures of ITPK1 orthologs [22, 38–40].

Relative to *At*ITPK4, ITPK1 possesses a ‘tether’ insertion following the central subdomain, while AtITPK4 possesses a ‘tab’ in the N-terminal sub-domain [22]. The tether comprises a polypeptide connection between the C-terminal β-strand of the central domain and a helix of the C-terminal domain running under the protein. This polypeptide lies across the top of the active site cavity linking the two domains (Figure 3B,D). In all the ITPK1s of known molecular structure where this polypeptide is resolved it provides residues that contribute to the ATP co-factor/substrate binding pocket. Consistent with the absence of resolved nucleotide, this region is disordered in the crystal structure of *Zm*ITPK1 [38] but in *St*ITPK1 it is stabilized in most part by adventitious interactions with a neighbouring copy of the molecule in the crystal lattice. Both the tab and tether insertions help shape the active site cleft in plant ITPK1 and ITPK4, suggesting they may contribute to differential substrate recognition (Figure 3B,C). As for other ITPK1 enzymes, the active site of *St*ITPK1 is narrow, unlike the more open active site in *At*ITPK4 (Figure S7), and features a highly positively charged active site (Figure 3D).

**Figure 4:**
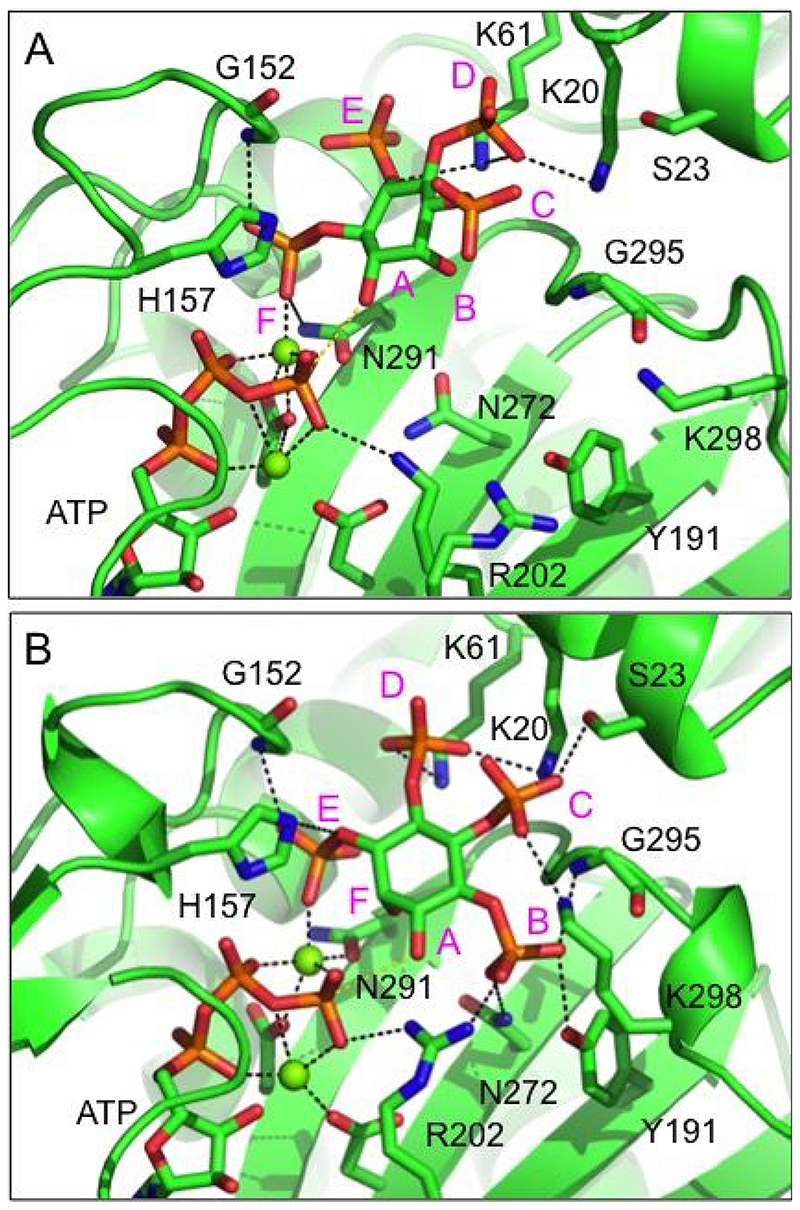
Prediction of the binding modes of an enantiomeric pair of substrates in the active site of *St*ITPK1. **A**, A closeup view of the energy minimized predicted binding mode of the poor substrate, Ins(1,4,5,6)P_4_, in the kinase domain active site. The enzyme is shown in cartoon format and coloured green. The substrate and active site residues (labelled) with which it forms polar interactions are shown in stick format with carbon coloured green, oxygen red, nitrogen blue and phosphorus orange. Magnesium ions are shown as dark green spheres. Polar interactions are indicated by black, dashed lines. Specificity subsites are labelled A-F (magenta font) such that the hydroxyl group positioned to accept the γ-phosphate of ATP by in-line transfer (the hydroxyl attached to carbon 3 of the inositol ring, in this case) occupies subsite A and the remaining subsites are arrayed in an anticlockwise sense when observed from the viewpoint adopted in this figure. **B**, View of the energy minimized predicted binding mode of the good substrate, Ins(3,4,5,6)P_4_, in the kinase domain active site. The hydroxyl attached to carbon 1 of the inositol ring, in this case occupies subsite A. Display format and colouring as in panel A.

To explain the preference of *St*ITPK1 for its substrates we modelled binding of the Ins(1,4,5,6)P_4_ and Ins(3,4,5,6)P_4_ enantiomers to *St*ITPK1, adopting the consensus specificity subsite nomenclature [39]. Briefly, subsite A is the site of phosphoryl transfer and constitutes the catalytic centre. For Ins(1,4,5,6)P_4_ (Figure 4A), substituents on locants 3, 2, 1, 6, 5 and 4 of the *myo*-inositol ring occupy sites A, B, C, D, E and F, respectively, while for Ins(3,4,5,6)P_4_ (Figure 4B), substituents on locants 1, 6, 5, 4, 3 and 2 occupy sites A, B, C, D, E and F, respectively. Residues forming polar interactions with the hydroxyl and phosphate groups of the substrates in the relaxed models are summarized (Table S2). The predicted pose of the poor substrate, Ins(1,4,5,6)P_4_, lacks polar interactions in the B- and C-subsites (Figure 4A), while the strong substrate, Ins(3,4,5,6)P_4_, enjoys polar interactions in all subsites except F, occupied by the 2-hydroxyl group (Figure 4B). In the B-pocket, the 6-phosphate of Ins(3,4,5,6)P_4_ is predicted to interact with the sidechain of Asn272. The substitution of a conserved glycine residue at this site in the ITPK4s (Gly437 in *At*ITPK4) may help to explain the poor activity of *At*ITPK4 towards this potential substrate. If accurately predicted, these interactions in the B- and C-subsites are likely crucial for enantiospecific hydroxy-kinase activity by *St*ITPK1 towards inositol tetrakisphosphates.

A model for a stereochemically-productive complex of InsP_6_ with *St*ITPK1 derived by molecular docking is shown in Figure S8. All residues observed to interact with InsP_6_ in its complex with the maize enzyme [38] are conserved in *St*ITPK1. However, due to the lack of bound nucleotide in the crystal structure of *Zm*ITPK1, the central domain is displaced relative to that seen in the potato enzyme structure and InsP_6_ binds in such a way that in-line phosphoryl transfer from the γ-phosphate of ATP is implausible [39, 41]. It therefore appears that the absence of nucleotide from the structure of the complex of *Zm*ITPK1 with InsP_6_ leads to a situation where an unproductive binding mode is stabilized. On the other hand, in the pose of InsP_6_ predicted for *St*ITPK1, InsP_6_ binds with its ‘receiving’ 5-phosphate in the F-specificity subsite (Figure S8), contrasting with the ‘receiving’ 1-hydroxyl of Ins(3,4,5,6)P_4_ which occupies subsite A (Figure 4). The minimum distance in this pose from the γ-phosphate phosphorus atom of ATP to a 5-phosphate oxygen of the substrate is a little over 3.5 Å rendering plausible in-line phosphoryl transfer from the γ-phosphate of ATP to generate the observed 5-PPInsP_5_ product. Again, polar contacts to the ligand are made by Q224 of the tether region, thus the bound nucleotide may stabilize the central subdomain and a portion of the tether, enabling recognition of InsP_6_ and catalysis. In *Zm*ITPK1, the tether is part of a catalytic specificity element that sanctions phospho-kinase activity against InsP6 [38]. The absence of the tether polypeptide in ITPK4 would then be consistent with its inability to synthesize inositol pyrophosphates [20, 22].

### PP-InsP analogs confirm the stereospecificity of *St*ITPK1 for phosphorylation of PP-InsPs

**Figure 5:**
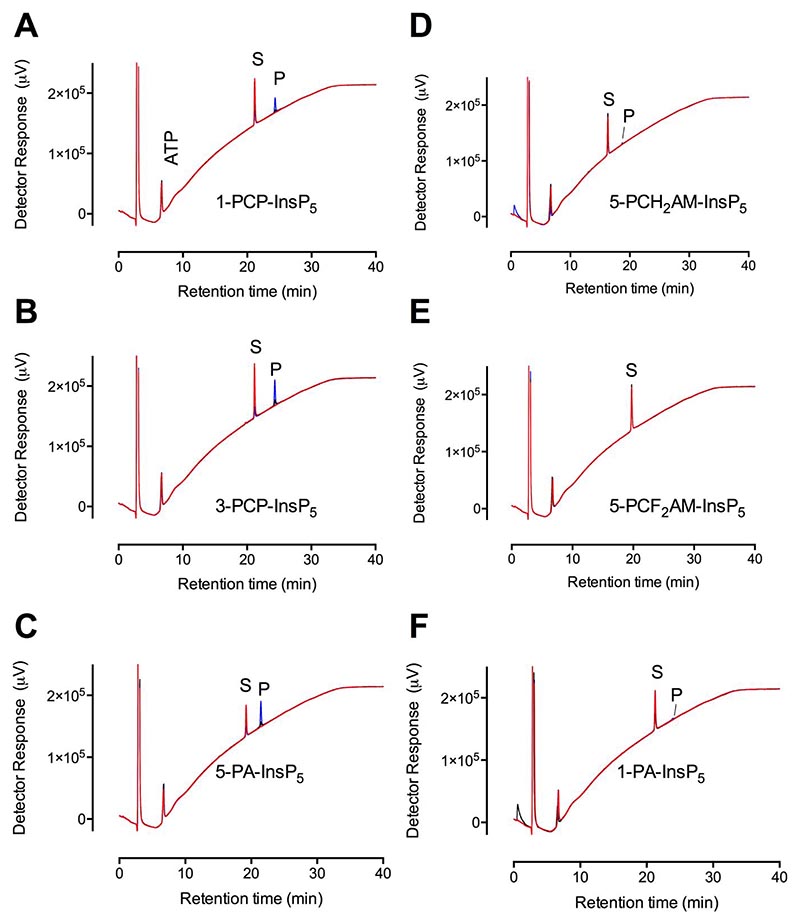
Phosphorylation of inositol pyrophosphate analogs by *St*ITPK1. HPLC resolution of products of reaction of *St*ITPK1 with ATP and **A**, 3-PCP-Ins(1,2,4,5,6)P_5_ [3-PCP-InsP_5_]; **B**, 1-PCP-Ins(2,3,4,5,6)P_5_ [1-PCP-InsP_5_]_;_ **C**, 5-PA-Ins(1,2,3,4,6)P_5_ [5-PA-InsP_5_]; **D**, 5-PCH_2_AM-Ins(1,2,3,4,6)P_5_ [5-PCH_2_AM-InsP_5_]; **E**, 5-PCF_2_AM-Ins(1,2,3,4,6)P_5_ [5-PCF_2_AM-InsP_5_]; **F**, 1-PA-Ins(2,3,4,5,6)P_5_ [1-PA-InsP_5_]. Substrates are indicated, S; products, P. For all panels, chromatograms of reactions without enzyme are shown in red, 3h incubations with enzyme are shown in black and 12h incubations in blue. The position of elution of ATP is shown in panel A. The HPLC column was eluted with a gradient of HCl.

For both *St*ITPK1 and *At*ITPK1, InsP_6_ was the strongest phospho-kinase substrate, with 3-PP-InsP_5_ a stronger substrate than 1-PP-InsP_5_, whereas 5-PP-InsP_5_ was not a substrate (Figure 1, Figure S4, Table S3). Nevertheless, the three PP-InsP_5_s tested showed similar *K*_i_ for displacement of 2-FAM-InsP_5_ from *St*ITPK1, in the range 88-178 nM (Figure S9A-C). Overall, PP-InsP_5_s displayed similar *K*_i_ to InsP_6_ isomers (cf. Figure 2 and Figure S9). We also tested a range of PP-InsP analogs as phospho-kinase substrates (Figure 5). Interestingly, both 3-PCP-Ins(1,2,4,5,6)P_5_, hereafter 3-PCP-InsP_5_, and 1-PCP-Ins(2,3,4,5,6)P_5_, hereafter 1-PCP-InsP_5_, were substrates. They yielded products, with identical retention times, that we assume to be 3-PCP-, 5-PP-Ins(1,2,4,6)P_4_ and 1-PCP-, 5-PP-Ins(2,3,4,6)P_4_ respectively (Figure 5A,B). *At*ITPK1 also displays the same preference for enantiomers of PCP-InsP_5_ analogs (Figure S4).

For both *St*ITPK1 and *At*ITPK1, 3-PCP-InsP_5_ and 1-PCP-InsP_5_ were better substrates than their ‘parents’ 3-PP-InsP_5_ and 1-PP-InsP_5_ (cf. Figure 5, Figure S4), though both compounds gave *K*_i_ for displacement of 2-FAM-InsP_5_ between 2 and 4-times higher than the ‘parents’ (Figure S9). For *St*ITPK1, 5-PA-Ins(1,2,3,4,6)P_5_, hereafter 5-PA-InsP_5_, was the strongest phospho-kinase substrate after *myo*-InsP_6_ (Figure 5C, Table S3), but was a weak substrate for *At*ITPK1 (Figure S4G). From this compound, both enzymes generated a single product that eluted shortly after *myo*-InsP_6_ and substantially before 5-PP-InsP_5_. Retention between InsP_6_ and 5-PP-InsP_5_ is indicative of pyrophosphorylation, with the non-reactive phosphono-acetoxy group (on the 5-position) likely contributing a little less to interaction with the column than a 5-phosphomonoester.

**Figure 6:**
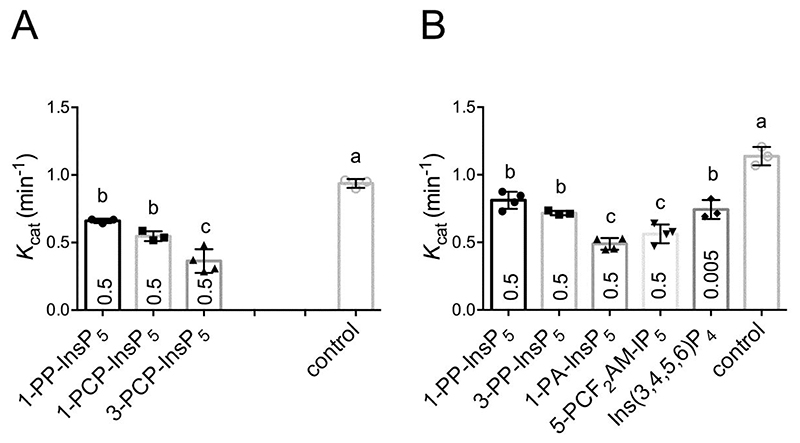
Inhibition of *St*ITPK1 phospho-kinase activity by inositol pyrophosphate analogs. **A**, Ins(1,2,3,4,5)P_5_ 5-phospho-kinase activity; **B**, InsP_6_ 5-phospho-kinase activity. Reactions were performed for 2h at 30 °C with 3 μM *St*ITPK1, 0.5 mM ATP, 1mM inositol phosphate in the absence or presence of competitor. The extent of inhibition at inhibitor concentration (mM) indicated by number in each column was estimated from the integrated peak areas of substrate and product peaks resolved by HPLC (example HPLC traces are shown in Figure S10). Significant difference at *p*=0.05 between inhibitor treatments (by One-way ANOVA and Tukey’s multiple comparisons test) is indicated by the absence of a common letter.

Of the other PP-InsP_5_ analogs, 5-PCH_2_AM-Ins(1,2,3,4,6)P_5_, hereafter 5-PCH_2_AM-InsP_5_, and 1-PA-Ins(2,3,4,5,6)P5, hereafter 1-PA-InsP_5_, were very weak substrates (Figure 5D,F), while 5-PCF_2_AM-Ins(1,2,3,4,6)P_5_, hereafter 5-PCF_2_AM-InsP_5_, was not a substrate (Figure 5E). 5-PCH_2_AM-InsP_5_ and 5-PCF_2_AM-InsP_5_ displaced 2-FAM-InsP_5_ from *St*ITPK1 with *K*_i_ of 889 nM and 106 nM, respectively (Figure S9E,F), while 1-PA-InsP_5_ displaced 2-FAM-InsP_5_ with a *K*_i_ of 300 nM. For 5-PCP-Ins(1,2,3,4,6)P_5_, hereafter 5-PCP-InsP_5_, a *K*_i_ of 101 nM was obtained (Figure S9D) and for 5-PP-InsP_5_ a *K*_i_ of 162 nM was obtained (Figure S9C). These observations are consistent with polar contacts for the bridging anhydride oxygen atom of 5-PP-InsP_5_ and the fluorine atom(s) of 5-PCF_2_AM-InsP_5_, these being absent for the hydride H atom(s) of 5-PCH_2_AM-InsP_5_. The electron-withdrawing effect of fluorine atoms might also be expected to increase the negative charge density of the terminal phosphonate in 5-PCF_2_AM-InsP_5_. Irrespective of whether molecules were substrates or not, inositol phosphates, inositol pyrophosphates and analogs, alike, were inhibitors of the Ins(1,2,3,4,5)P_5_ phospho-kinase activity of *St*ITPK1 with inhibition for this assay falling in the range 42-89% (Figure 6, Table S3).

Example HPLC traces showing reduced generation of 5-PP-InsP_4_ product from Ins(1,2,3,4,5)P_5_ on inclusion of 1-PCP-InsP_5_ or 3-PCP-InsP_5_ are presented (Figure S10). Both 1-PCP-InsP_5_ and 3-PCP-InsP_5_ are potent inhibitors (Figure 6, Figure S10, Table S3), confirming the predictive power of the fluorescence polarization assays which yielded a lower *K*_i_ for 3-PCP-InsP_5_ than for 1-PCP-InsP_5_, both lower than for Ins(1,2,3,4,5)P_5_ (cf. Figure 2D, Figure S9G,H). Consistent with this, the 3-PCP-analog was also the more potent inhibitor of Ins(1,2,3,4,5)P_5_ phospho-kinase activity (Figure 6A, Figure S10B,C, Table S3). In the absence of inhibitor, turnover of Ins(1,2,3,4,5)P_5_ and InsP_6_ were similar giving *K*_cat_ of 0.94±0.03 min^-1^ and 1.14±0.07 min^-1^. Of all the compounds tested against InsP_6_ (Figure 6B, and Table S3), Ins(3,4,5,6)P_4_, *K*_i_ 716 nM, was a particularly powerful inhibitor of the inositol pyrophosphate- (5-PP-InsP_5_) synthesizing activity of *St*ITPK1, even at a concentration, 5 μM, that is 2-orders of magnitude lower than the concentration of InsP_6_ substrate. These data are consistent with the previous study of AtITPK1 [21], in which *V*_max_ with Ins(3,4,5,6)P_4_ was 3 orders of magnitude greater than for InsP_6_. These data provide a mechanism by which Ins(3,4,5,6)P_4_ can regulate the phospho-kinase activity of ITPK1. We speculate that Ins(3,4,5,6)P_4_ competes with InsP_6_ as substrate, both are found in plants [4,15,30] where Ins(3,4,5,6)P_4_ is a precursor of InsP_6_ [30,31]. We further suggest that enhanced accumulation of Ins(3,4,5,6)P_4_ in *itpk1* mutants [9,15,27] and *ipk1* mutants [4,15] amplifies reductions in PP-InsP species [9,15,27] that are widely reported to be the agents of much physiology [3]. Indeed, *itpk1* and *ipk1* mutants show a constitutive phosphate starvation response [4,14,15].

## Discussion

Our understanding of inositol pyrophosphate function rests heavily on molecular genetic disruption of hydroxy-kinase and phospho-kinase activities. These have pleiotropic influence on plant physiology, reflecting involvement in processes as diverse as pathogen resistance, symbiosis, phosphate starvation response and the action of plant growth regulators auxin and jasmonate. Disruption also has multifaceted effect on inositol phosphate metabolism, with impacts on ‘lower’ and ‘higher’ inositol phosphates and inositol pyrophosphates alike. This is particularly apparent for ITPKs [9, 15, 27] and IP5K (IPK1) [4, 15].

Ablation of *At*ITPK1 increases lower inositol phosphates [9, 15, 27] and reduces PP-InsP_5_ and [PP]_2_-InsP_4_ alike, in plants. While it has been widely assumed that [PP]_2_-InsP_4_-synthesizing activity belongs exclusively to VIH1/2 [3, 6, 15, 23–24, 42], until recent detailed description of non-canonical PP-InsP_5_ species [9, 21, 27] little consideration had been given to other possibilities. Similarly, rather little consideration has been given to the possibility that different inositol phosphates and inositol pyrophosphates are active site competitors of ATP-grasp kinases. In respect of the latter premise, the data presented here suggests that competition could be significant and the principle likely applies to other ATP-grasp kinases. In respect of the former premise, the null hypothesis, that ITPK1 does not contribute directly (ie., other than by provision of 5-PP-InsP_5_ substrate to VIH1/2) to [PP]_2_-InsP_4_ levels, is challenged. The pronounced inhibition of phospho-kinase activity by Ins(3,4,5,6)P_4_ says as much, while the reversibility of IPK1’s phospho-kinase activity (previously described for InsP_6_ substrate [21,27], here tested against 1-PP-InsP_5_ and 3-PP-InsP_5_) extends ITPK1 influence to whole cohorts of inositol pyrophosphate substrates that are widely reported in plants [1–3, 6–10, 15, 23–24, 27, 34, 42]. Taken together, the foregoing raises the possibility of adenine-nucleotide- (energy charge) dependent substrate-cycles between [PP]_2_-InsP_4_ and PP-InsP_5_ species, offering extra dimension to the central role of ITPK1 in diverse physiological phenomena [3–10]. Nonetheless, recent work has also identified a family of Plant and Fungi Atypical Dual Specificity Phosphatases (PFA-DSPs) that display activity against multiple inositol pyrophosphates. In vitro, they, like *At*ITPK1 and *St*ITPK1 here, show specificity for removal of the β-5-phosphate of 5-PP-InsP_5_, 1,5-[PP]_2_-InsP_4_ and 3,5-[PP]_2_-InsP_4_ alike [34].

By corollary, it seems plausible that VIH1/2 also show reversible phospho-kinase (ie. ATP-synthesizing) activity: not only do VIH1/2 possess the ATP-grasp fold, but the mammalian homolog PPIP5K2 shows ADP-driven 1,5-[PP]_2_-InsP_4_ 1-dephosphorylation (ATP-synthesizing) activity [28, 43]. It is, perhaps, instructive therefore to compare the relative activities of different ATP-grasp kinase proteins of plants. *At*VIH2 has been characterized as separate kinase and phosphatase domains [24]. The former shows 1-phospho-kinase activity against InsP_6_ and 5-PP-InsP_5_ with turnover numbers of ~0.4 and ~1 (min^-1^) respectively [24]. These constants were derived under assay conditions very similar to those employed in the present and earlier studies of *At*ITPK1 [21,27]. *K*_cat_ for *At*ITPK1’s phospho-kinase activity against InsP_6_, calculated from data [20–21, 27] is comparable, ~1.5 min^-1^, here ~1.14 min^-1^ at pH 6.5, while *K*_cat_ for 5-PP-InsP_5_-driven ATP synthesis is greater [27]. Clearly, energy charge acts through ITPK1 and other ATP-grasp kinases to modulate the PP-InsP_5_s and [PP]_2_-InsP_4_(s) that are widely reported to influence plant physiology. Indeed, genetic evidence shows that despite these very low *K*_cat_ values for both *At*ITPK1 and *At*VIH2, ablation of *At*ITPK1 markedly reduces the levels of PP-InsP_5_ and [PP]_2_-InsP_4_ in plants [9,27], while ablation of VIH1/2 markedly reduces [PP]_2_-InsP_4_ [8,24]. The low cellular levels of these species, relative to InsP_6_, typically less than 1%, perhaps implies that the bulk of cellular InsP_6_ is accessible to *At*ITPK1. The difference in rate constants for 5-pyrophosphorylation and hydroxy-kinase activities may arise from poorer geometry for the former, with placement of the receiving 5-phosphate or hydroxyl (for the latter) in different enzyme subsites.

## Conclusions

The catalytic flexibility of the ATP-grasp fold kinase ITPK1 has been extended to include inositol pyrophosphates considered previously to be substrates/products only of VIH1/2, among kinases. Detailed analysis of binding of diverse substrates and substrate analogs to *St*ITPK1 was enabled by the use of fluorescence polarization assays. This assay may find use for other ATP-grasp kinases. This and the solution of a nucleotide-liganded crystal structure for *St*ITPK1 offer opportunity for more precise elaboration of inositol pyrophosphate function in plants, one that accommodates competition by substrates/inhibitors for cognate partners such as TIR1, COI1-ASK and COP signalosome components and enzymes alike. Plant ITPK1 also offers opportunity for study of ATP-grasp kinases in pyrophosphate biology contexts by provision of probes of phosphotransfer. One envisages the simple incorporation of ^32^P or ^33^P from γ-labeled ^32^/^33^P ATP into the β-5-phosphate of 1,5-[PP]_2_-InsP_4_ and 3,5-[PP]_2_-InsP_4_ and analogs thereof.

## Supplementary Material

Supplementary data

## Abbreviations

*At*ITPK, IP5K: *Arabidopsis thaliana* inositol pentakisphosphate 2-kinase
*At*ITPK1: *Arabidopsis thaliana* inositol tris/tetrakisphosphate kinase 1
*At*ITPK4: *Arabidopsis thaliana* inositol tris/tetrakisphosphate kinase 4
D-*chiro*-InsP_6_: 1-D- *chiro*-inositol 1,2,3,4,5,6-hexakisphosphate
EDTA: ethylenediamine tetra-acetic acid
HEPES: 4-(2-hydroxyethyl)-1-piperazineethane sulfonic acid
His: histidine
HPLC: high-pressure liquid chromatography
*Eh*ITPK1: *Entamoeba histolytica* inositol tris/tetrakisphosphate kinase 1
*Hs*ITPK1: *Homo sapiens* inositol tris/tetrakisphosphate kinase 1
IP6K: inositol hexakisphosphate kinase
Ins(1,4,5,6)P_4_: 1D-*myo*-inositol 1,4,5,6-tetrakisphosphate; 1D-*myo*-inositol 2,3,4,6-tetrakisphosphate
Ins(3,4,5,6)P_4_: 1D-*myo*-inositol 3,4,5,6-tetrakisphosphate
Ins(1,2,3,4,5)P_5_: 1D-*myo*-inositol 1,2,3,4,5-pentakisphosphate
Ins(1,2,3,5,6)P_5_: 1D-*myo*-inositol 1,2,3,5,6-pentakisphosphate
Ins(1,2,4,5,6)P_5_, InsP_6_, Ins(1,2,3,4,5,6)P_6_: *myo*-inositol 1,2,3,4,5,6-hexakisphosphate
3-PP-Ins(1,2,4,5)P_4_: 1D-3-diphospho-*myo*-inositol 1,2,4,5-tetrakisphosphate
5-PP-Ins(1,2,3,4)P_4_: 1D-5-diphospho-*myo*-inositol 1,2,3,4-tetrakisphosphate
1-InsP_7_, 1-PP-InsP_5_: 1D-1-diphospho-*myo*-inositol 2,3,4,5,6-pentakisphosphate
2-FAM-InsP_5_: 2-O-(2-(5-fluoresceinylcarboxy)-aminoethyl)-*myo*-inositol 1,3,4,5,6-pentakisphosphate (triethylammonium salt)
3-InsP7, 3-PP-InsP_5_: 1D-3-diphospho-*myo*-inositol 1,2,4,5,6-pentakisphosphate
4-InsP7, 4-PP-InsP_5_: 1D-4-diphospho-*myo*-inositol 1,3,5,6-pentakisphosphate
5-InsP_7_, 5-PP-InsP_5_: 5-diphospho-*myo*-inositol 1,2,3,4,6-pentakisphosphate
6-InsP_7_, 6-PP-InsP_5_: 1D-6-diphospho-*myo*-inositol 1.2.3.4.5-pentakisphosphate
InsP_8_: bis-diphospho-*myo*-inositol-tetrakisphosphate
1,5-InsP_8_: 1D-1,5-bis-diphospho-*myo*-inositol 2,3,4,6-tetrakisphosphate
3,5-InsP_8_: 1D-3,5-bis-diphospho-*myo*-inositol 1,2,4,6-tetrakisphosphate
MES: 2-(N-morpholino)ethanesulfonic acid
*neo*-InsP_6_: *neo*-inositol 1,2,3,4,5,6-hexakisphosphate
NMR: Nuclear Magnetic Resonance Spectroscopy
OPLS: optimized potentials for liquid simulations
PAGE: Polyacrylamide Gel Electrophoresis
PCR: polymerase chain reaction
PDB: Protein DataBank
PEG 6000: polyethylene glycol 6000
PPIP5K: diphosphoinositol pentakisphosphate kinase
*rac*-InsP_8_: 1:1 mixture of 1,5-InsP_8_ and 3,5-InsP_8_
*scyllo*-InsP_6_;: *scyllo*-inositol 1,2,3,4,5,6-hexakisphosphate
*St*ITPK1: *Solanum tuberosum* inositol tris/tetrakisphosphate kinase 1
TLS: Translation-Libration-Screw-rotation
TLSMD, TLS: Translation-Libration-Screw Motion Determination
VIH1: *Arabidopsis thaliana* diphosphoinositol pentakisphosphate kinase 1
VIH2: *Arabidopsis thaliana* diphosphoinositol pentakisphosphate kinase 2
VSGB: Generalized Born continuum solvent model
*Zm*ITPK1: *Zea mays* inositol tris/tetrakisphosphate kinase 1
1-PCP-InsP_5_: *myo*-inositol 2,3,4,5,6-penta-phosphate-1-methylenediphosphonate
3-PCP-InsP_5_: *myo*-inositol 1,2,4,5,6-penta-phosphate-3-methylenediphosphonate
5-PA-InsP_5_: *myo*-inositol 1,2,3,4,6-penta-phosphate-5-phosphonoacetate
5-PCH_2_Am-InsP_5_: 5-deoxy-5-(phosphonoacetamido)-*myo*-inositol 1,2,3,4,6-pentakisphosphate
5-PCF_2_Am-InsP_5_: 5-deoxy-5-(phosphonodifluoroacetamido)-*myo*-inositol 1,2,3,4,6-pentakisphosphate
5-PCP-InsP_5_: *myo*-inositol 1,2,3,4,6-penta-phosphate-5-methylenediphosphonate
1-PCP-,5-PP-Ins(2,3,4,6)P_4_: 5-diphospho-*myo*-inositol 2,3,4,6-tetra-phosphate-1-methylenediphosphonate
3-PCP-,5-PP-Ins(1,2,4,6)P_4_: 5-diphospho-*myo*-inositol 1.2.4,6-tetra-phosphate-3-methylenediphosphonate
