# Supplementary material for: Crystal Structure and Enzymology of Solanum tuberosum Inositol Tris/Tetrakisphosphate Kinase 1 (StITPK1)
Source: Biochemistry. 2023 Dec 26;63(1):42–52. doi: 10.1021/acs.biochem.3c00404 (PMC10765375; doi:10.1021/acs.biochem.3c00404)
Supplement: Supplementary file 1 [file bi3c00404_si_001.pdf]

Supporting Information

Crystal structure and enzymology of *Solanum tuberosum* inositol tris/tetrakisphosphate kinase 1 (*St*ITPK1)

Hayley L. Whitfield[1], Raquel Faba Rodríguez[1,2], Megan L. Shipton[3], Arthur W.H. Li[1,2], Andrew M. Riley[3], Barry V.L. Potter[3], *Andrew M. Hemmings[1,2,4] and *Charles A. Brearley[1]

[1]School of Biological Sciences, University of East Anglia, Norwich Research Park, Norwich NR4 7TJ, U.K.

[2]School of Chemistry, University of East Anglia, Norwich Research Park, Norwich NR4 7TJ, U.K.

[3]Medicinal Chemistry & Drug Discovery, Department of Pharmacology, University of Oxford, Mansfield Road, Oxford OX1 3QT, UK

[4]College of Food Science and Technology, Shanghai Ocean University, Shanghai 201306, China

*Correspondence: Charles Brearley: c.brearley@uea.ac.uk and Andrew Hemmings: a.hemmings@uea.ac.uk

Materials and methods

References

Supplementary Figures S1 - S10.

Supplementary Tables S1 - S3

Abbreviations

MATERIALS AND METHODS

Reagents

2-FAM-$InsP_5$ was synthesized as described [1]. Inositol phosphates and assay reagents for enzyme assays were obtained from sources described [2-4]. Among these, the $InsP_6$ isomers were obtained from the soil-extracted collections of the late Dennis Cosgrove and Max Tate. $InsP_8$ (1,5-$[PP]_2$-$InsP_4$) and *rac*-$InsP_8$ (racemic-1,5-$[PP]_2$-$InsP_4$) were synthesized using methodology developed for 5-PP-$InsP_5$ [5], employing intermediates described in [6]. These were purified by ion-exchange chromatography and thoroughly characterized by NMR and mass spectrometry. They were >95% pure and were used as their triethylammonium salts. Example syntheses of inositol pyrophosphate analogs used have been described previously [5-12].

Protein expression and purification

AtITPK1 was prepared as described [2]. StITPK1 was cloned initially as described [13]. Recombinant *St*ITPK1 was produced in *Escherichia coli* fused to an N-terminal hexahistidine tag and purified but this full-length form failed to crystallize even after removal of the His-tag. A disorder prediction study generated by the Protein DisOrder prediction System (PrDOS) [14] using a prediction false positive rate set at 5.0 % identified a region of disorder at the C-terminus. The Alphafold Protein Structure Database [15] confirmed this prediction. Consequently, a new construct was designed, again with an N-terminal His-tag, but in a truncated form bearing residues 8-320 of *St*ITPK1. This deletion construct was cloned by PCR into a pOPINF plasmid and the expression construct verified by sequencing. Expression and purification were carried out according to a previously published protocol including removal of the N-terminal His-tag by 3C cleavage [2].

X-ray crystal structure determination

Purified truncated *St*ITPK1 to which had been added a 4-fold molar excess of ADP was concentrated to 12.5 mg/mL. Single crystals were grown using the sitting drop vapour diffusion method by equilibration at 16 °C

against a crystallization solution containing 0.2 M calcium chloride dihydrate, 0.1 M MES pH 6.0 and 20% (w/v) PEG 6000. A single crystal was harvested into a cryoprotect solution containing 30% (v/v) ethylene glycol and X-ray diffraction data collected at 100 °C on beamline i24 at the Diamond Light Source (Oxford). Crystals were of the orthorhombic space group C222, with cell parameters a = 89.44 Å, b = 140.64 Å, c = 55.58 Å; $\alpha = \beta = \gamma = 90°$. Molecular replacement phasing was performed with Phaser [16] and the crystal structure of *Zm*ITPK1 (PDB: 7TN5) [17] as a search model. Extensive manual rebuilding using Coot [18] interspersed with restrained refinement with Phenix.refine [19-20] was necessary to complete the final structural model. Refinement employed a TLS model generated by the TLSMD web server [21-22]. The C-terminal 45 amino acids and residues 211-214 and 227-233 were found to be disordered and are omitted from the structure.

Molecular modelling

The procedure employed was based on that used previously to predict the binding of substrates to the crystal structure of *At*ITPK4 [3]. Molecular models of the complexes formed between *St*ITPK1 and an enantiomeric pair of inositol tetrakisphosphates were generated with reference to the crystal structure of the *Entamoeba histolytica* ITPK1 (*Eh*ITPK1) in complex with Mg^{2+}/AMP-PCP/Ins(1,3,4)P_3 (PDB entry 1Z2P). AMP-PCP, phosphomethylphosphonic acid adenylate ester, is a non-hydrolyzable ATP analogue. Least squares superposition of the β-sheet residues of the C-terminal domains of *Eh*ITPK1 from PDB entry 1Z2P and *St*ITPK1 produced, by direct transfer of atomic coordinates, a draft docking pose of Ins(1,3,4,6)P_4 and the ATP analogue in the active site of the potato enzyme. ATP coordinates were substituted for AMP-PCP and subsequent *in silico* substitution of phosphate groups of the inositol polyphosphate ligand generated models of, in turn, Ins(1,4,5,6)P_4 or Ins(3,4,5,6)P_4. Rotation of the ligands 180° about the C2-C5 axis (where Cn indicates the carbon number, n, of the inositol ring) followed by rotation about an axis normal to the ring produced models of Ins(1,4,5,6)P_4 or Ins(3,4,5,6)P_4 bound to *St*ITPK1 positioned for stereochemically-favoured hydroxy-kinase action at the C3 or C1 hydroxyl of the substrate, respectively [23]. A model for the docked complex of InsP_6 with *St*ITPK1 was predicted using the Glide module of Schrodinger (Schrödinger Release 2022-2: Glide,

Schrödinger, LLC, New York, NY, 2021). All models of the complexes were subsequently energy minimized to convergence using the Prime module of Schrodinger (Schrödinger Release 2022-2: Prime) employing the all atom OPLS force field [24-25] and the VSGB 2.1 implicit solvent model [26]. Illustrations were generated using the PyMOL Molecular Graphics System, Version 2.5 (Schrödinger, LLC).

HPLC analyses

Kinase assays were performed with ATP-regeneration as described for ATP-grasp kinases ITPK1/2 and VIH1/2 [2-3, 27-29], here in 20 mM HEPES pH 6.5, 1 mM $MgCl_2$, 0.5 mM ATP and 1 mM inositol phosphate substrate. The low rate constants for phospho-kinase activity and the low sensitivity of NMR, PAGE and HPLC assays demand long incubation times with high, but non-inhibitory concentrations of phospho-kinase substrates [2-3, 27-29], here with or without 4 µM protein for 3h or 12h at 25 °C. Competition/inhibition assays were performed under ATP-regenerating conditions with 0.5 mM ATP, 1 mM substrate, either Ins(1,2,3,4,5)P_5 or $InsP_6$, and competitor, typically 0.5 mM, incubated with or without 3 µM protein for 2 h at 25 °C. Reverse (phosphotransfer to ADP) assays were performed with 20 mM HEPES pH 6.5, 1 mM $MgCl_2$, 0.5mM ADP and 0.5 mM inositol pyrophosphate substrate incubated with 4 µM protein at 25 °C. HPLC analysis and detection of inositol phosphates by complexation with ferric ion was performed as described [2]. Peak area data were analysed with the chromatography Jasco ChromNav v.2 software and *x,y* data were imported into GraphPad Prism v.6 software (GraphPad Software Inc., San Diego, USA) for plotting of chromatograms without additional processing of the data.

Fluorescence polarization (anisotropy)

A curve for binding of 2-FAM-$InsP_5$ to *St*ITPK1 was obtained by incubation of 2 nM 2-FAM-$InsP_5$ [1] with increasing concentration (1-500 nM) of *St*ITPK1 in 20 mM HEPES pH 6.5, 1 mM EDTA, 100 mM NaCl, after [30]. Thereafter, displacement assays were performed by titration of inositol phosphate, inositol pyrophosphate

or analog thereof with 2 nM 2-FAM-$InsP_5$ and 50 nM *St*ITPK1. Compounds were tested for displacement in a range of 1 nM - 50 μM. Fluorescence polarization was measured in 384-well, black, low-binding plates, Corning product 3573. Binding and displacement curves (anisotropy) were log transformed and fitted to a 4-parameter logistic (in GraphPad Prism v.6 software (GraphPad Software Inc., San Diego, USA). Fitting of the binding of 2-FAM-$InsP_5$ to a specific one-site binding model in GraphPad Prism yielded a K_d of 48 nM. Substituting the value of K_d for K_M in the Cheng-Prusoff equation for competitive inhibition:

$$IC50 = Ki(1 + [S]/Km)$$

with concentration of 2-FAM-$InsP_5$ [S], yielded K_i with confidence intervals adjusted by linear approximation.

REFERENCES

1. Watson, P. J.; Millard, C. J.; Riley, A. M.; Robertson, N. S.; Wright, L. C.; Godage, H. Y.; Cowley, S. M.; Jamieson, A. G.; Potter, B. V. L.; Schwabe, J. W., Insights into the activation mechanism of class I HDAC complexes by inositol phosphates. *Nat Commun* 2016, *7*, 11262.

2. Whitfield, H.; White, G.; Sprigg, C.; Riley, A. M.; Potter, B. V. L.; Hemmings, A. M.; Brearley, C. A.; An ATP-responsive metabolic cassette comprised of inositol tris/tetrakisphosphate kinase 1 (ITPK1) and inositol pentakisphosphate 2-kinase (IPK1) buffers diphosphosphoinositol phosphate levels. *Biochemical Journal* 2020, *477*, 2621-2638.

3. Whitfield, H. L.; He, S.; Gu, Y.; Sprigg, C.; Kuo, H-F.; Chiou, T. J.; Riley, A. M.; Potter, B. V. L.; Hemmings, A. M.; Brearley, C. A., Diversification in the inositol tris/tetrakisphosphate kinase (ITPK) family: crystal structure and enzymology of the outlier *At*ITPK4. *Biochem J* 2023, *480* (6), 433-453.

4. Whitfield, H.; Riley, A. M.; Diogenous, S.; Godage, H. Y.; Potter, B. V. L.; Brearley, C. A., Simple synthesis of (32)P-labelled inositol hexakisphosphates for study of phosphate transformations. *Plant Soil* **2018,** *427* (1-2), 149-161.

5. Riley, A. M.; Wang, H.; Weaver, J. D.; Shears, S. B.; Potter, B. V. L., First synthetic analogues of diphosphoinositol polyphosphates: interaction with PP-InsP5 kinase. *Chem Commun (Camb)* 2012, *48* (92), 11292-4.

6. Brown, N W.; Marmelstein, A. M.; Fiedler, D., Chemical tools for interrogating inositol pyrophosphate structure and function. *Chem Soc Rev* 2016, *45* (22), 6311-6326.

7. Riley, A. M.; Wang, H.; Shears, S. B.; Potter, B. V. L., Synthetic tools for studying the chemical biology of $InsP_8$. *Chem Commun (Camb)* 2015, *51* (63), 12605-8.

8. Wu, M.; Dul, B. E.; Trevisan, A. J.; Fiedler, D., Synthesis and characterization of non-hydrolysable diphosphoinositol polyphosphate second messengers. *Chem Sci* 2013, *4* (1), 405-410.

9. Wu, M.; Chong, L. S.; Capolicchio, S.; Jessen, H. J.; Resnick, A. C.; Fiedler, D., Elucidating diphosphoinositol polyphosphate function with nonhydrolyzable analogues. *Angew Chem Int Ed Engl* 2014, *53* (28), 7192-7.

10. Hager, A.; Wu, M.; Wang, H.; Brown, N. W., Jr.; Shears, S. B.; Veiga, N.; Fiedler, D., Cellular Cations Control Conformational Switching of Inositol Pyrophosphate Analogues. *Chemistry* 2016, *22* (35), 12406-14.

11. Riley, A. M.; Wang, H.; Shears, S. B.; Potter, B. V. L., Synthesis of an alpha-phosphono-alpha,alpha-difluoroacetamide analogue of the diphosphoinositol pentakisphosphate 5-InsP(7). *Medchemcomm* 2019, *10* (7), 1165-1172.

12. Wang, H.; Godage, H. Y.; Riley, A. M.; Weaver, J. D.; Shears, S. B.; Potter, B. V. L, Synthetic inositol phosphate analogs reveal that PPIP5K2 has a surface-mounted substrate capture site that is a target for drug discovery. *Chem Biol* 2014, *21* (5), 689-99.

13. Caddick, S. E.; Harrison, C. J.; Stavridou, I.; Mitchell, J. L.; Hemmings, A. M.; Brearley, C. A., A *Solanum tuberosum* inositol phosphate kinase (StITPK1) displaying inositol phosphate-inositol phosphate and inositol phosphate-ADP phosphotransferase activities. *FEBS Lett* 2008, *582* (12), 1731-7.

14. Ishida, T.; Kinoshita, K., PrDOS: prediction of disordered protein regions from amino acid sequence. *Nucleic Acids Res* 2007, *35* (Web Server issue), W460-4.

15. Varadi, M.; Anyango, S.; Deshpande, M.; Nair, S.; Natassia, C.; Yordanova, G.; Yuan, D.; Stroe, O.; Wood, G.; Laydon, A.; Zidek, A.; Green, T.; Tunyasuvunakool, K.; Petersen, S.; Jumper, J.; Clancy, E.; Green, R.; Vora, A.; Lutfi, M.; Figurnov, M.; Cowie, A.; Hobbs, N.; Kohli, P.; Kleywegt, G.; Birney, E.; Hassabis, D.; Velankar, S., AlphaFold Protein Structure Database: massively expanding the structural coverage of protein-sequence space with high-accuracy models. *Nucleic Acids Res* 2022, *50* (D1), D439-D444.

16. McCoy, A. J.; Grosse-Kunstleve, R. W.; Adams, P. D.; Winn, M. D.; Storoni, L. C.; Read, R. J., Phaser crystallographic software. *J Appl Crystallogr* 2007, *40* (Pt 4), 658-674.

17. Zong, G.; Shears, S. B.; Wang, H., Structural and catalytic analyses of the InsP6 kinase activities of higher plant ITPKs. *FASEB J* 2022, *36* (7), e22380.

18. Emsley, P.; Lohkamp, B.; Scott, W. G.; Cowtan, K., Features and development of Coot. *Acta Crystallogr D Biol Crystallogr* 2010, *66* (Pt 4), 486-501.

19. Adams, P. D.; Afonine, P. V.; Bunkoczi, G.; Chen, V. B.; Davis, I. W.; Echols, N.; Headd, J. J.; Hung, L. W.; Kapral, G. J.; Grosse-Kunstleve, R. W.; McCoy, A. J.; Moriarty, N. W.; Oeffner, R.; Read, R. J.;

Richardson, D. C.; Richardson, J. S.; Terwilliger, T. C.; Zwart, P. H., PHENIX: a comprehensive Python-based system for macromolecular structure solution. *Acta Crystallogr D Biol Crystallogr* 2010, *66* (Pt 2), 213-21.

20. Afonine, P. V.; Grosse-Kunstleve, R. W.; Echols, N.; Headd, J. J.; Moriarty, N. W.; Mustyakimov, M.; Terwilliger, T. C.; Urzhumtsev, A.; Zwart, P. H.; Adams, P. D., Towards automated crystallographic structure refinement with phenix.refine. *Acta Crystallogr D Biol Crystallogr* 2012, *68* (Pt 4), 352-67.

21. Painter, J.; Merrit, E.A., TLSMD web server for the generation of multi-group TLS models. *J Appl Crystallogr* 2006, *39*, 109-111.

22. Painter, J.; Merritt, E. A., Optimal description of a protein structure in terms of multiple groups undergoing TLS motion. *Acta Crystallogr D Biol Crystallogr* 2006, *62* (Pt 4), 439-50.

23. Riley, A. M.; Deleu, S.; Qian, X.; Mitchell, J.; Chung, S. K.; Adelt, S.; Vogel, G.; Potter, B. V. L.; Shears, S. B., On the contribution of stereochemistry to human ITPK1 specificity: Ins(1,4,5,6)P4 is not a physiologic substrate. *FEBS Lett* 2006, *580* (1), 324-30.

24. Jorgensen, W. L.; Tirado-Rives, J., The OPLS [optimized potentials for liquid simulations] potential functions for proteins, energy minimizations for crystals of cyclic peptides and crambin. *J Am Chem Soc* 1988, *110* (6), 1657-66.

25. Harder, E.; Damm, W.; Maple, J.; Wu, C.; Reboul, M.; Xiang, J. Y.; Wang, L.; Lupyan, D.; Dahlgren, M. K.; Knight, J. L.; Kaus, J. W.; Cerutti, D. S.; Krilov, G.; Jorgensen, W. L.; Abel, R.; Friesner, R. A., OPLS3: A Force Field Providing Broad Coverage of Drug-like Small Molecules and Proteins. *J Chem Theory Comput* 2016, *12* (1), 281-96.

26. Li, J.; Abel, R.; Zhu, K.; Cao, Y.; Zhao, S.; Friesner, R. A., The VSGB 2.0 model: a next generation energy model for high resolution protein structure modeling. *Proteins* 2011, *79* (10), 2794-812.

27. Laha, D.; Parvin, N.; Hofer, A.; Giehl, R. F. H.; Fernandez-Rebollo, N.; von Wiren, N.; Saiardi, A.; Jessen, H. J.; Schaaf, G., Arabidopsis ITPK1 and ITPK2 Have an Evolutionarily Conserved Phytic Acid Kinase Activity. *ACS Chem Biol* 2019, *14* (10), 2127-2133.

28. Zhu, J.; Lau, K.; Puschmann, R.; Harmel, R. K.; Zhang, Y.; Pries, V.; Gaugler, P.; Broger, L.; Dutta, A. K.; Jessen, H. J.; Schaaf, G.; Fernie, A. R.; Hothorn, L. A.; Fiedler, D.; Hothorn, M., Two bifunctional inositol pyrophosphate kinases/phosphatases control plant phosphate homeostasis. *Elife* 2019, *8*.

29. Riemer, E.; Qiu, D.; Laha, D.; Harmel, R. K.; Gaugler, P.; Gaugler, V.; Frei, M.; Hajirezaei, M. R.; Laha, N. P.; Krusenbaum, L.; Schneider, R.; Saiardi, A.; Fiedler, D.; Jessen, H. J.; Schaaf, G.; Giehl, R. F. H., ITPK1 is an $InsP_6$/ADP phosphotransferase that controls phosphate signaling in Arabidopsis. *Mol Plant* 2021, *14* (11), 1864-1880.

30. Whitfield, H.; Gilmartin, M.; Baker, K.; Riley, A. M.; Godage, H. Y.; Potter, B. V. L.; Hemmings, A. M.; Brearley, C. A.; A Fluorescent Probe Identifies Active Site Ligands of Inositol Pentakisphosphate 2-Kinase. *Journal of Medicinal Chemistry* 2018, *61*, 8838-8846.

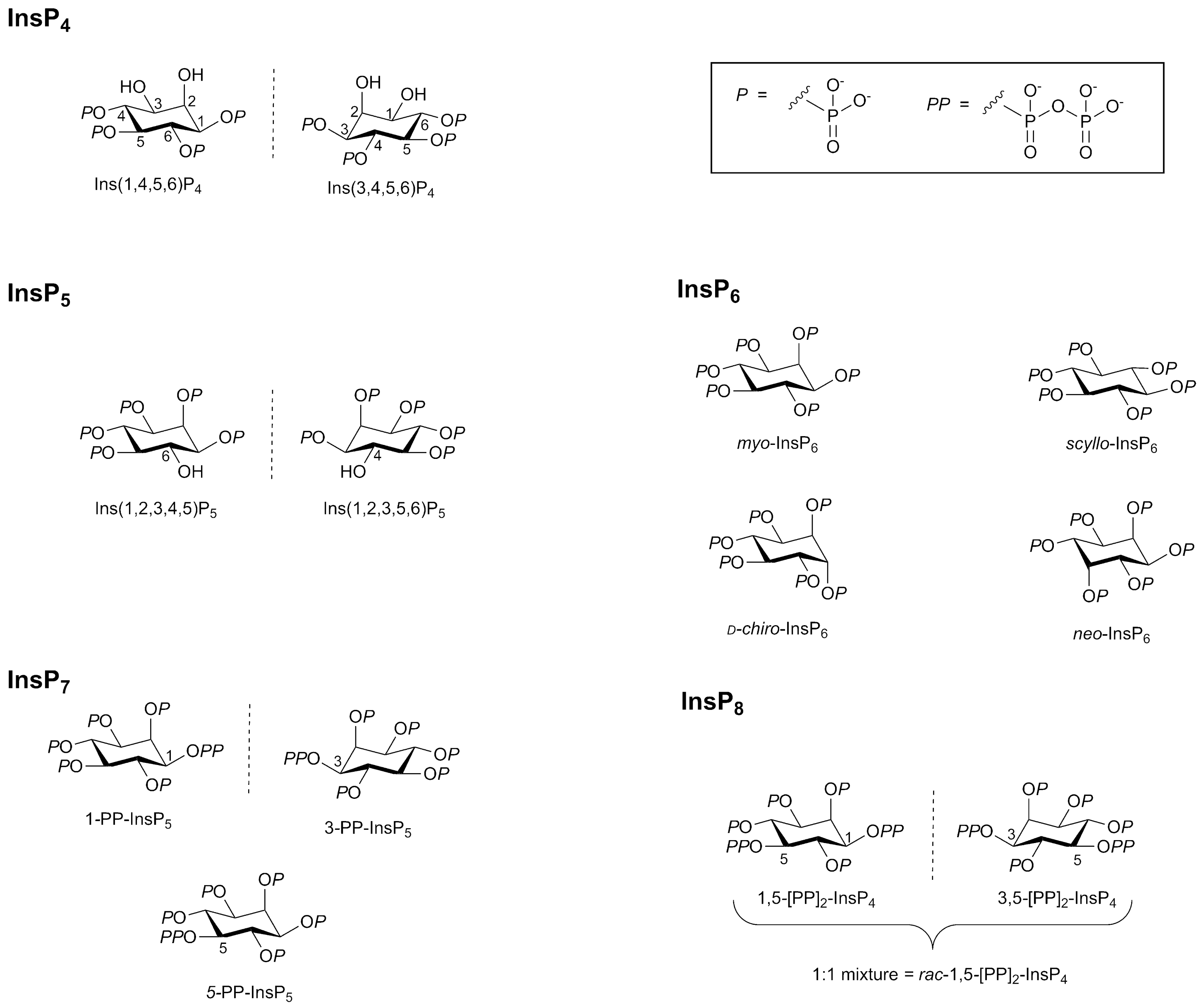

Figure S1. Structures of inositol phosphates, inositol pyrophosphates and analogs tested as substrates or ligands of *St*ITPK1. Enantiomeric pairs are shown reflected in a plane of symmetry.

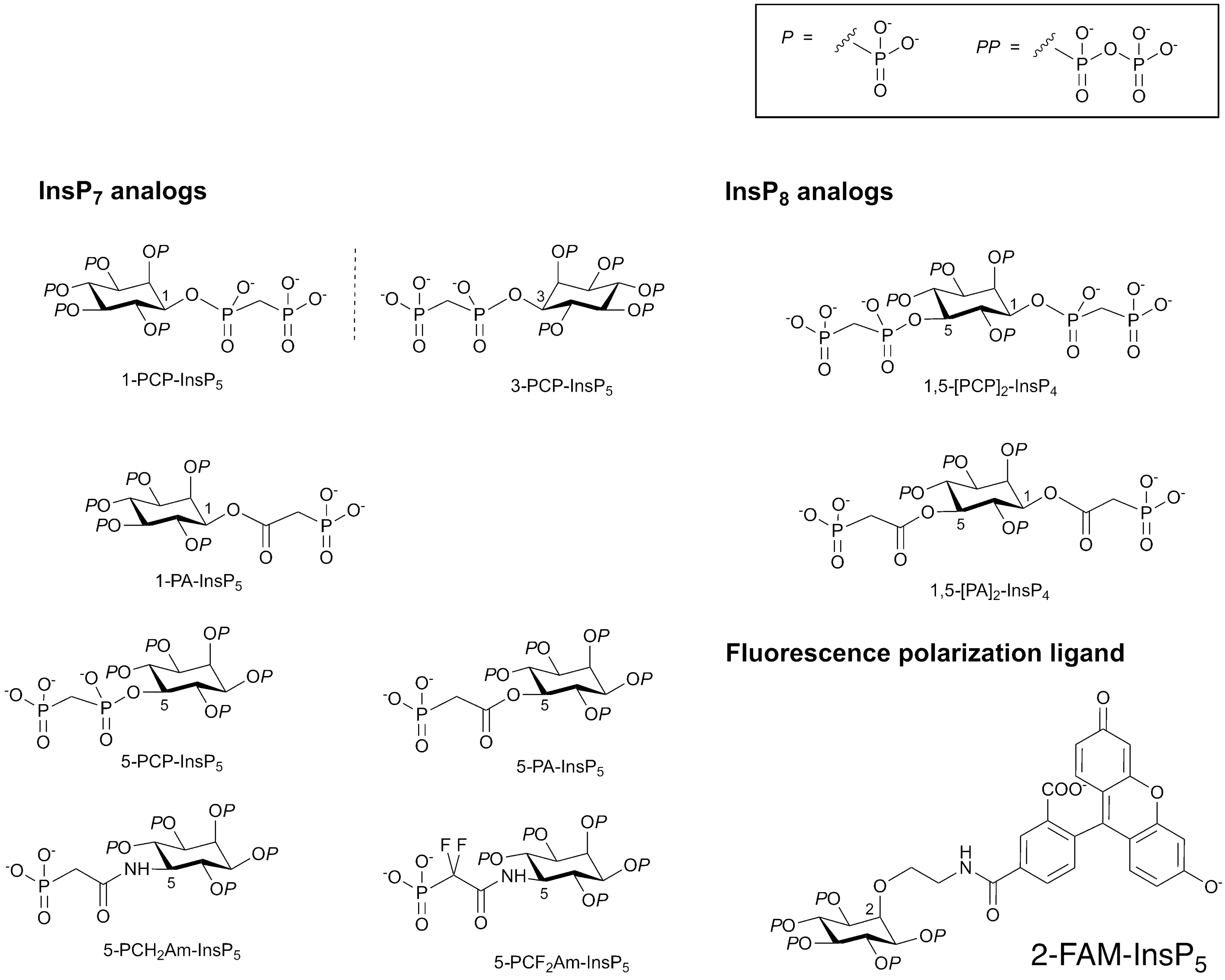

Figure S1. Structures of inositol phosphates, inositol pyrophosphates and analogs tested as substrates or ligands of *St*ITPK1 continued. Enantiomeric pairs are shown reflected in a plane of symmetry.

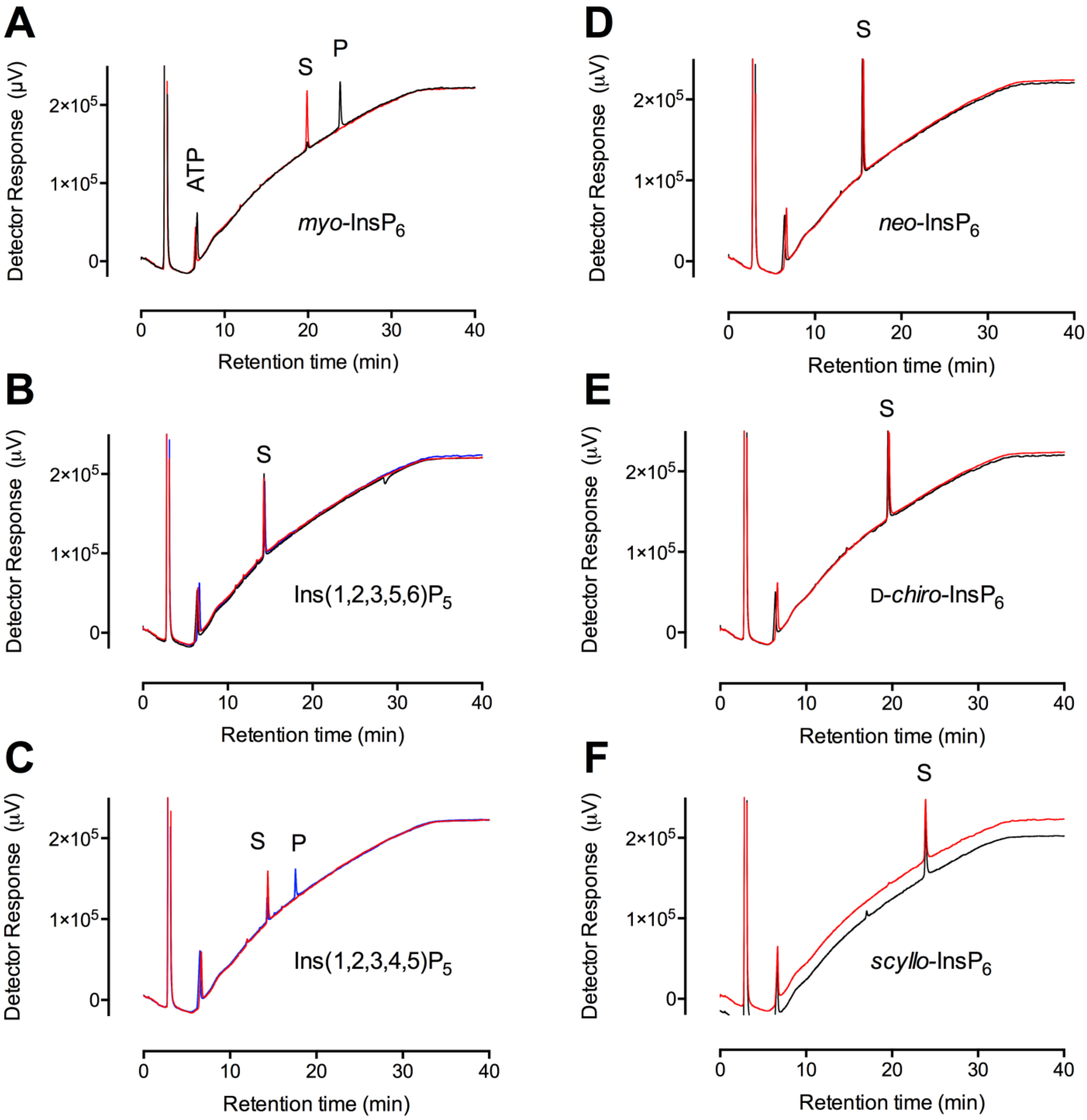

Figure S2. *St*ITPK1 displays 5-phosphokinase activity towards higher *myo*-inositol phosphate substrates. HPLC resolution of products of 12h reaction of *St*ITPK1 with ATP and **A**, *myo*-InsP$_6$; **B**, Ins(1,2,3,5,6)P$_5$; **C**, Ins(1,2,3,4,5)P$_5$; **D**, *neo*-InsP$_6$; **E**, D-*chiro*-InsP$_6$, **F**, *scyllo*-InsP$_6$. Substrates are indicated, S; products, P. Chromatograms of reactions without enzyme are shown in red and those with enzyme in black. A standard of 5-PP-InsP$_5$ (in buffer components lacking enzyme) is shown offset on the *y*-axis in blue, in A. The HPLC column was eluted with a gradient of HCl. The position of elution of ATP is shown in panel A.

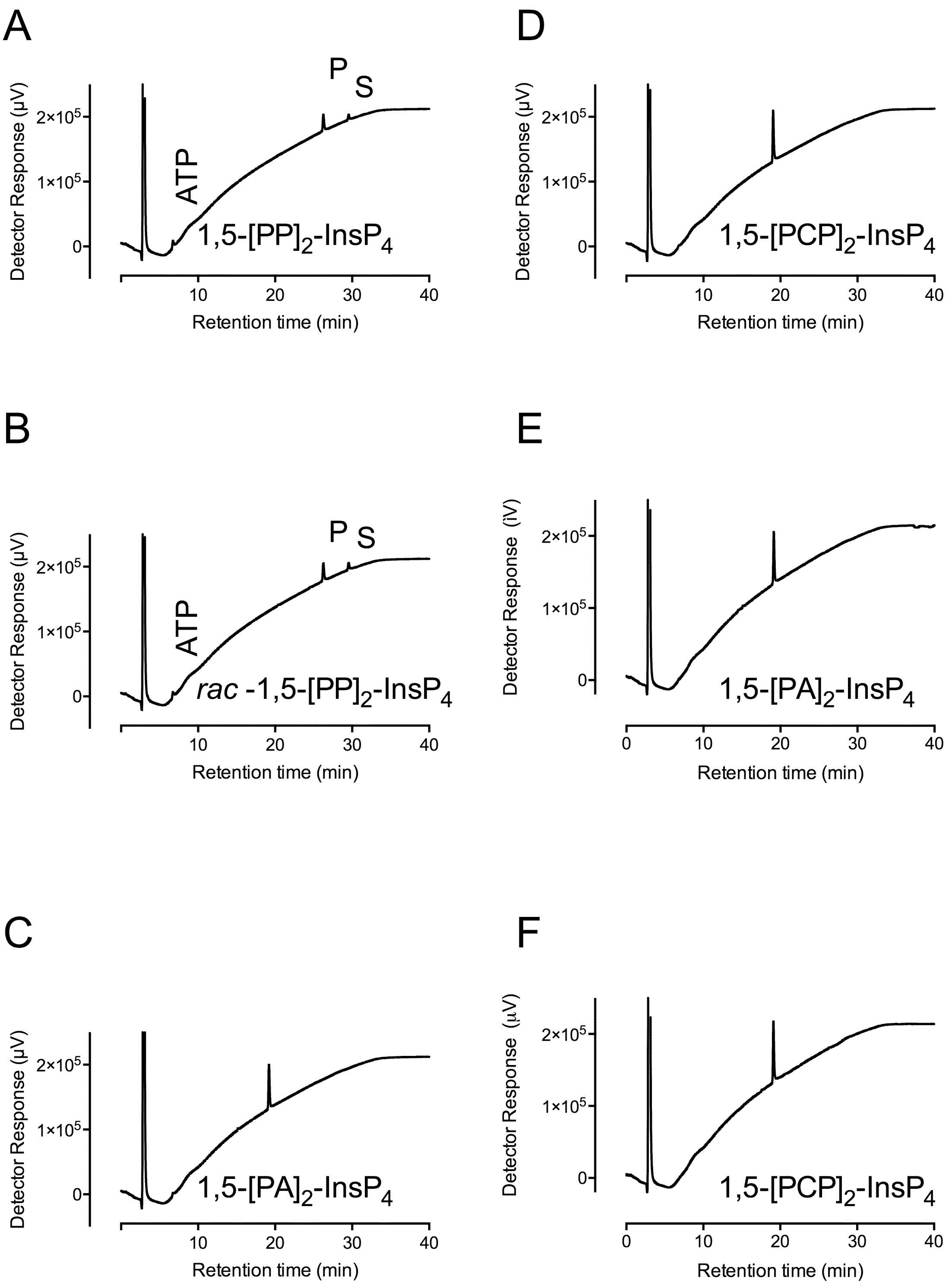

Figure S3 *At*ITPK1 is an inositol pyrophosphate-ADP phosphotransferase. HPLC of products of reaction of *At*ITPK1 with ADP and **A**, 1,5-[PP]$_2$-InsP$_4$; **B**, *rac*-1,5-[PP]$_2$-InsP$_4$; **C**, 1,5-[PA]$_2$-InsP$_4$; **D**, 1,5-[PCP]$_2$-InsP$_4$. Substrates are indicated, S; products, P. The position of elution of ATP formed by phosphotransfer to ADP is shown in panels A, B. ADP elutes in the solvent front. Note the absence of ATP peaks in C and D. HPLC of products of 12h reaction of *St*ITPK1 with ADP and **E**, 1,5-[PA]$_2$-InsP$_4$; **F**, 1,5-[PCP]$_2$-InsP$_4$. The HPLC column was eluted with a gradient of HCl.

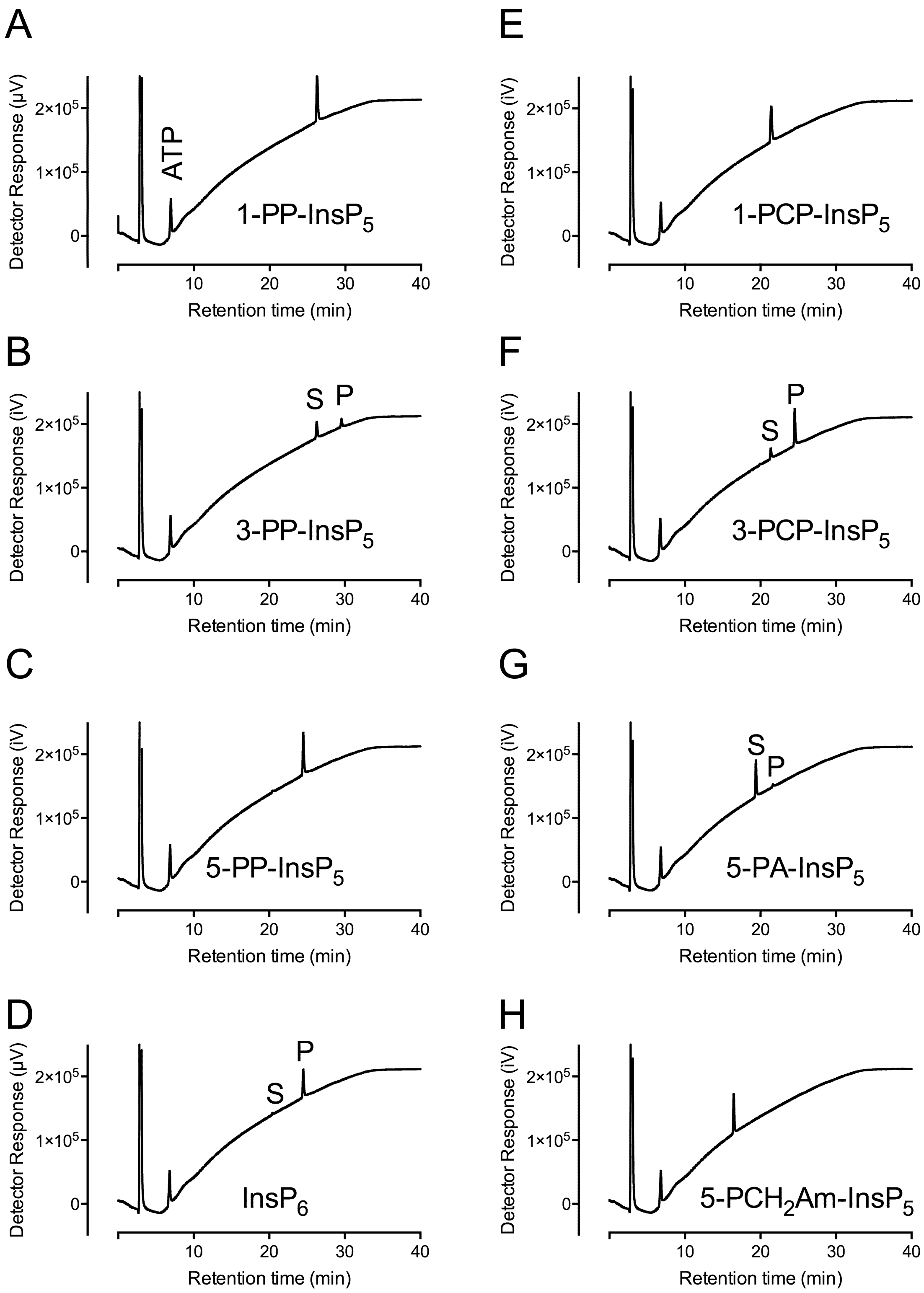

Figure S4. *At*ITPK1 is an inositol pyrophosphate phosphokinase. HPLC of products of reaction of *At*ITPK1 with ATP and **A**, 1-PP-$InsP_5$; **B**, 3-PP-$InsP_5$; **C**, 5-PP-$InsP_5$; **D**, $InsP_6$; **E**, 1-PCP-$InsP_5$; **F**, 3-PCP-$InsP_5$; **G**, 5-PA-$InsP_5$; **H**, 5-PCH_2Am-$InsP_5$. Substrates are indicated, S; products, P. The position of elution of ATP is shown in panel A. The HPLC column was eluted with a gradient of HCl.

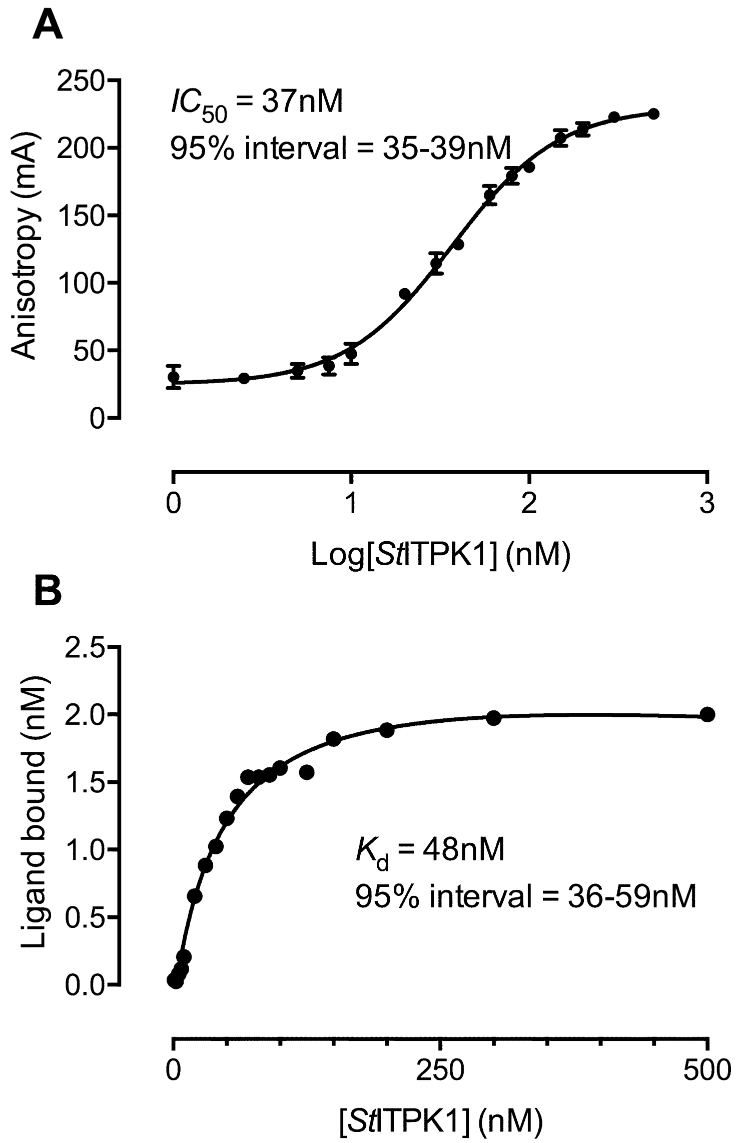

Figure S5. Binding of 2-FAM-InsP$_5$ to *St*ITPK1. **A**, 4-parameter logistic fit of anisotropy data. **B**, A fit of data of A to a one-site binding model. Data are the means and standard deviations of four replicate measurements.

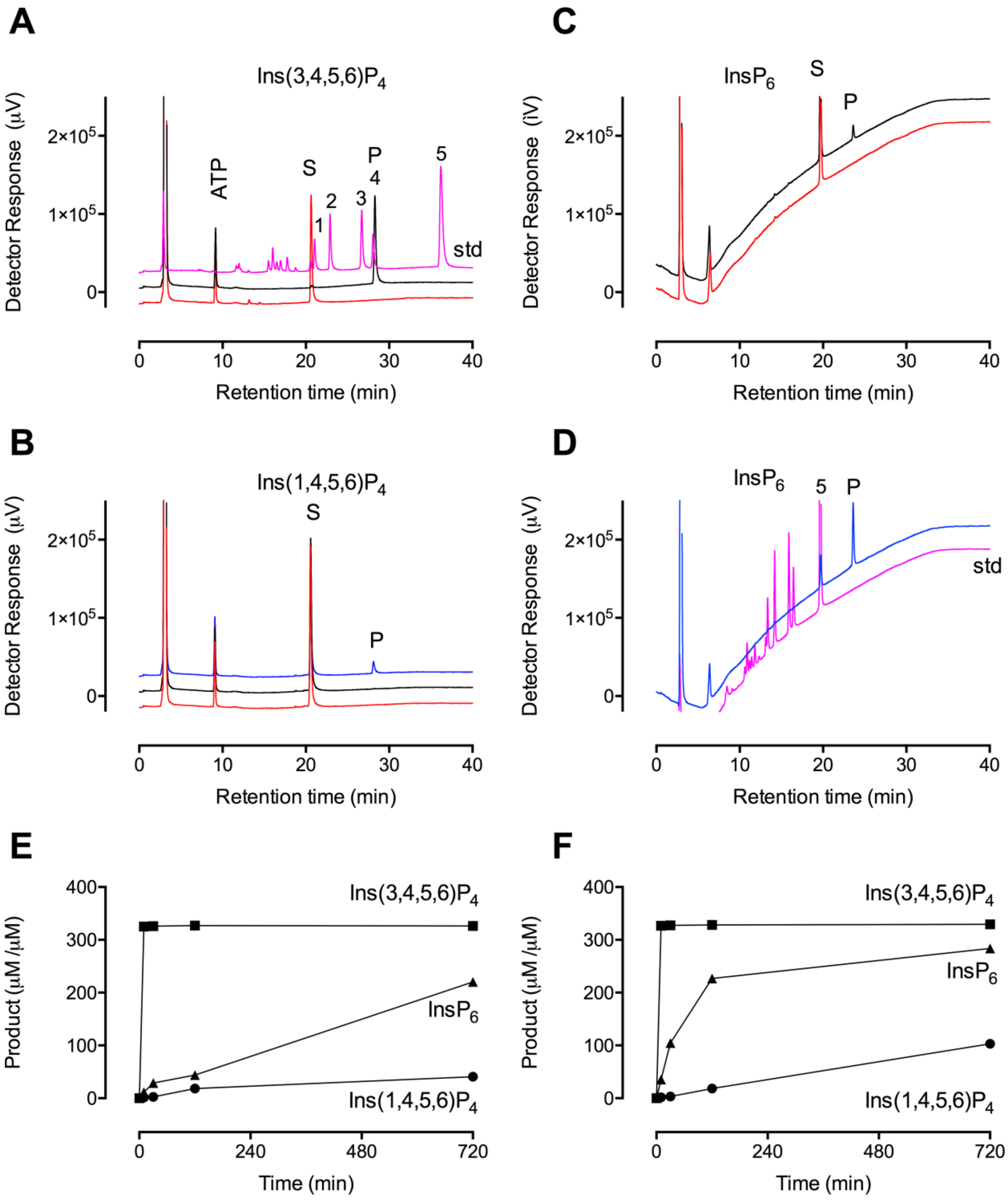

Figure S6. Enantiospecificity of *St*ITPK1 and *At*ITPK1 for Ins(3,4,5,6)P_4. HPLC of reactions of *St*ITPK1 with **A**, Ins(3,4,5,6)P_4; **B**, Ins(1,4,5,6)P_4; **C**, **D**, *myo*-InsP_6. Progress of reaction curves are shown **E**, for *St*ITPK1; **F**, for *At*ITPK1. Products were eluted with methanesulfonic acid, A and B; HCl, C and D, without enzyme (red); 10 min reaction (black) and 12h reaction (blue). Substrates are indicated, S, and products, P. Peaks among the standards (std) (magenta), an acid hydrolysate of InsP_6, include: 1, Ins(1,2,3,4,6)P_5; 2, Ins(1,2,3,4,5)P_5/Ins(1,2,3,5,6)P_5; 3, Ins(1,2,4,5,6)P_5/Ins(2,3,4,5,6)P_5; 4, Ins(1,3,4,5,6)P_5 and 5, InsP_6. The position of elution of ATP is shown in panels A and C. Pairwise comparison of reactivity between substrates has been performed for *St*ITPK1 more than 5 times with similar results. The data for *At*ITPK1 are confirmatory of detailed experiments described in reference [21].

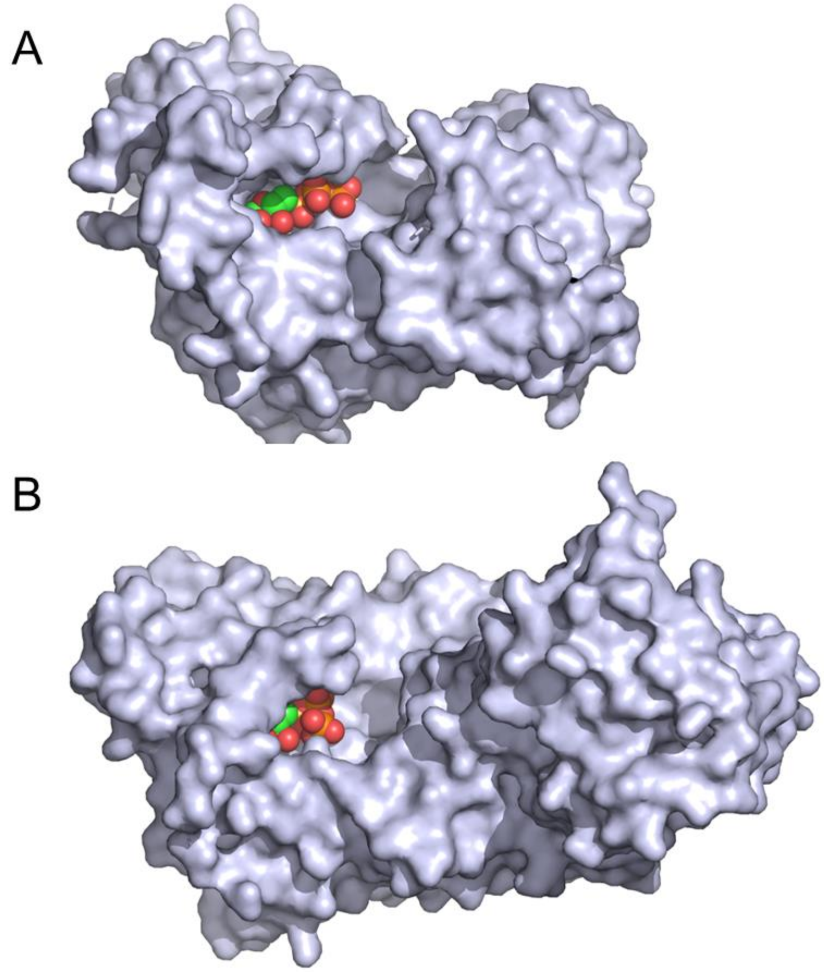

Figure S7. View onto the active site cavities of *St*ITPK1 and *At*ITPK4. **A**, A molecular surface representation of the structure of *St*ITPK1. **B**, A molecular surface representation of the structure of *At*ITPK4 (PDB: 7PUP). In both panels bound ADP/ATP is shown in atom sphere format with colouring: carbon-green, oxygen-red, nitrogen-blue and phosphorus-orange.

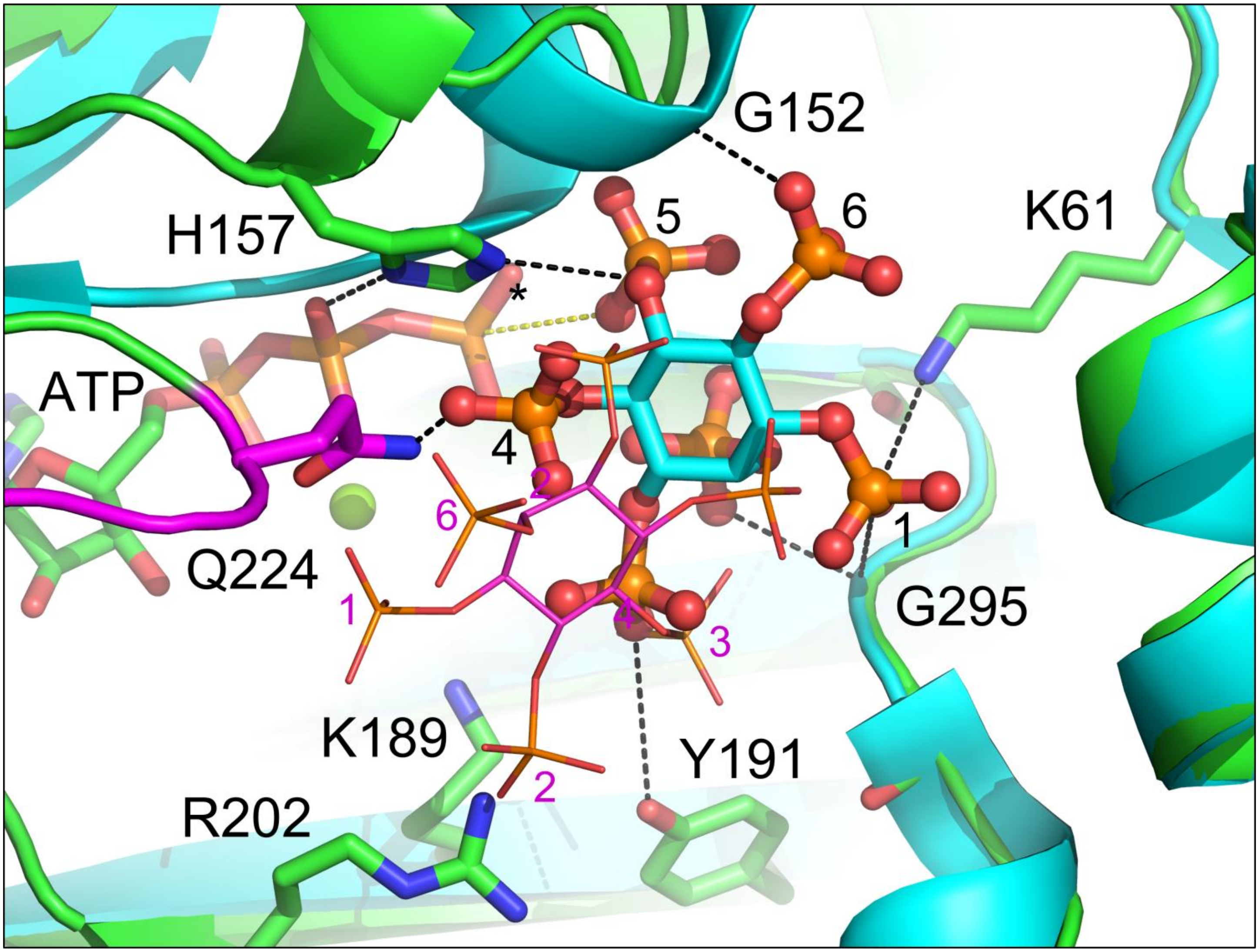

Figure S8. View of a predicted pose for binding of $InsP_6$ to *St*ITPK1. $InsP_6$ and residues including ATP within 4 Å are shown in stick format. The phosphates of the inositol ring of $InsP_6$ are numbered. Black dashed lines indicate putative polar interactions between the ligand and protein in the model. An asterisk indicates the vector (shown as a yellow dashed line) between the γ-phosphate phosphorus of ATP and the closest 5-phosphate oxygen of $InsP_6$. This distance is 3.5 Å in the model. Bond colouring is as follows: carbon-green (except of $InsP_6$ - cyan), oxygen-red, nitrogen-blue and phosphorus-orange. A cartoon representation of the fold of *St*ITPK1 is shown in green except for the start of the tether polypeptide, which is coloured magenta. For comparison, the equivalent pose of $InsP_6$ as seen in the crystal structure of its complex with *Zm*ITPK1 (PDB: 7TN8) is shown in line format with carbon atoms coloured magenta, oxygen-red and phosphorus-orange. Where it does not impact on clarity, the phosphate groups are labelled for reference. A cartoon representation of the fold of *Zm*ITPK1 is shown in cyan. Note that the absence of bound nucleotide in the *Zm*ITPK1 structure leads to a distinctly different positioning of the central domain relative to that seen in the complex of *St*ITPK1 with ADP (this study).

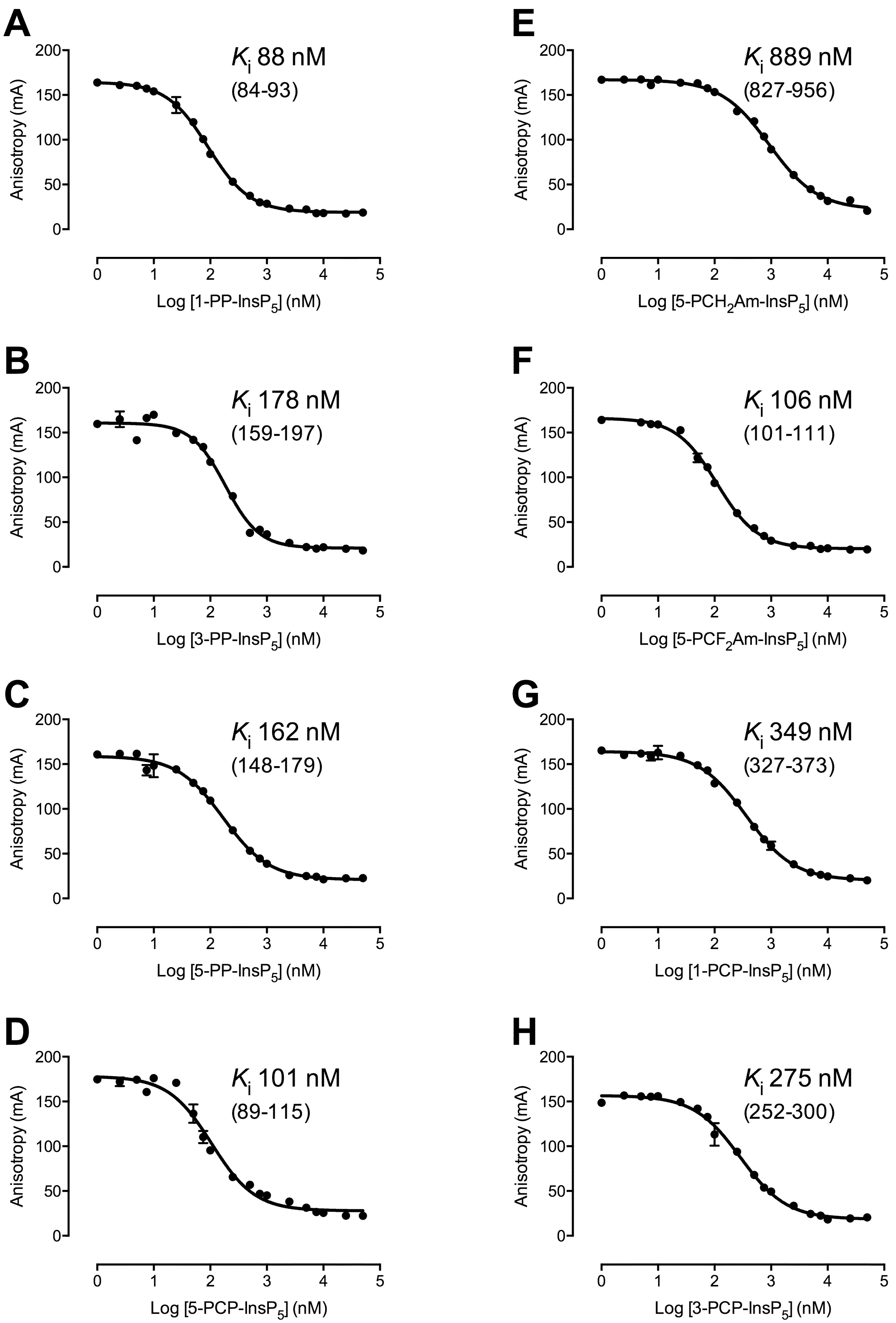
A
K_i 88 nM
(84-93)
Anisotropy (mA)
Log [1-PP-InsP$_5$] (nM)
B
K_i 178 nM
(159-197)
Anisotropy (mA)
Log [3-PP-InsP$_5$] (nM)
C
K_i 162 nM
(148-179)
Anisotropy (mA)
Log [5-PP-InsP$_5$] (nM)
D
K_i 101 nM
(89-115)
Anisotropy (mA)
Log [5-PCP-InsP$_5$] (nM)
E
K_i 889 nM
(827-956)
Anisotropy (mA)
Log [5-PCH$_2$Am-InsP$_5$] (nM)
F
K_i 106 nM
(101-111)
Anisotropy (mA)
Log [5-PCF$_2$Am-InsP$_5$] (nM)
G
K_i 349 nM
(327-373)
Anisotropy (mA)
Log [1-PCP-InsP$_5$] (nM)
H
K_i 275 nM
(252-300)
Anisotropy (mA)
Log [3-PCP-InsP$_5$] (nM)

Figure S9. High-affinity binding of inositol pyrophosphates and analogs to *St*ITPK1. Displacement of 2-FAM-InsP_5 with **A**, 1-PP-Ins(2,3,4,5,6)P_5 [1-InsP_7]; **B**, 3-PP-Ins(1,2,4,5,6)P_5 [3-InsP_7] **C**, 5-PP-Ins(1,2,3,4,6)P_5 [5-InsP_7]; **D**, 5-PCP-Ins(1,2,3,4,6)P_5 [5-PCP-InsP_5]; **E**, 5-PCH_2Am-Ins(1,2,3,4,6)P_5 [5-PCH_2Am-InsP_5]; **F**, 5-PCF_2-Ins(1,2,3,4,6)P_5 [5-PCF_2-InsP_5]; **G**, 1-PCP-Ins(1,2,4,5,6)P_5 [3-PCP-InsP_5]; **H**, 3-PCP-Ins(2,3,4,5,6)P_5 [1-PCP-InsP_5]. Data are the means and standard deviations of four replicates; K_i (nM) and confidence interval (nM) in parentheses.

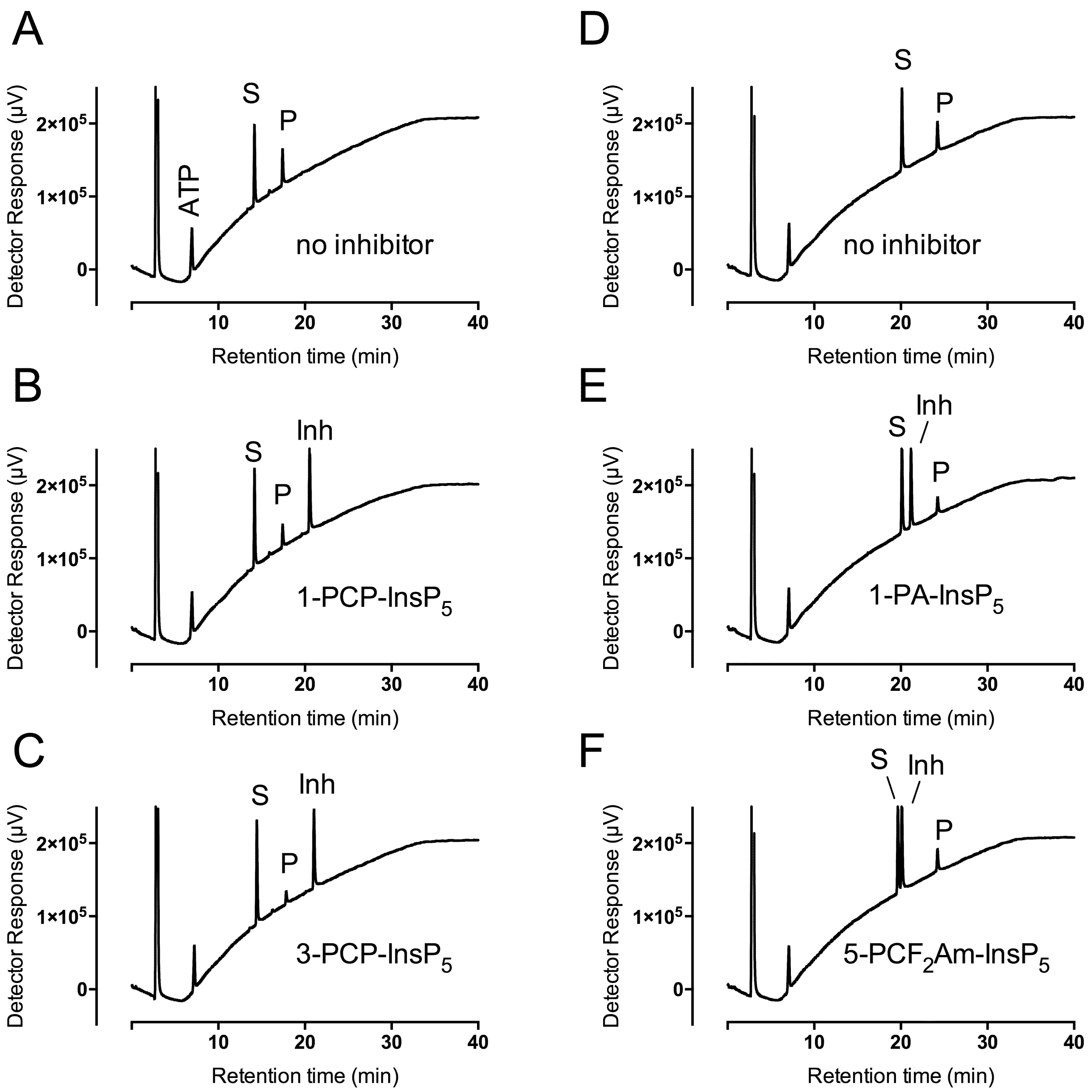

Figure S10. Inhibition of *St*ITPK1 phosphokinase activity by inositol pyrophosphate analogs. HPLC resolution of products of reaction of 3 μM *St*ITPK1 with ATP (0.5 mM) and either Ins(1,2,3,4,5)P_5 or $InsP_6$ (1.0 mM). Reactions were performed for 2h at 30 °C in the absence or presence of competitor (0.5 mM). **A**, Ins(1,2,3,4,5)P_5 substrate; **B**, Ins(1,2,3,4,5)P_5 substrate and 1-PCP-$InsP_5$; **C**, Ins(1,2,3,4,5)P_5 substrate and 3-PCP-$InsP_5$; **D**, $InsP_6$ substrate; **E**, $InsP_6$ substrate and 1-PA-$InsP_5$; **F**, $InsP_6$ substrate and 5-PCF_2Am-$InsP_5$. Substrates are indicated, S; products, P; and inhibitor, Inh. The position of elution of ATP is shown in panel A. The HPLC column was eluted with a gradient of HCl.

Table S1. Data collection and refinement statistics[a].

DATA COLLECTION	
Wavelength / Å	0.9686
Resolution range	70.39 - 2.26 (2.34 - 2.26)
Space group	C 2 2 2
Unit cell	89.6 140.8 55.6
Total reflections	377443 (26394)
Unique reflections	16840 (1640)
Multiplicity	22.4 (16.1)
Completeness (%)	99.69 (99.21)
Mean I/sigma(I)	20.82 (4.53)
Wilson B-factor	27.95
R-merge	0.2041
R-meas	0.2088 (0.993)
R-pim	0.04339 (0.2476)
CC1/2	0.998 (0.868)
CC*	1 (0.964)
REFINEMENT	
Reflections used in refinement	16835 (1641)
Reflections used for R-free	811 (70)
R-work	0.1897 (0.2344)
R-free	0.2411 (0.2779)
CC(work)	0.953 (0.880)
CC(free)	0.973 (0.834)
Number of non-hydrogen atoms	2598
macromolecules	2444
ligands	30
solvent	124
Protein residues	302
RMS(bonds)	0.01
RMS(angles)	1.23
Ramachandran favored (%)	95.95
Ramachandran allowed (%)	3.04
Ramachandran outliers (%)	1.01
Rotamer outliers (%)	2.22
Clashscore	7.46
Average B-factor	37.41
macromolecules	37.69
ligands	23.15
solvent	35.32
Number of TLS groups	4

Statistics for the highest-resolution shell are shown in parentheses.

Table S2. Predicted polar interactions (≤ 3.1 Å) with bound substrates in energy minimized inositol tetrakisphosphate models. A list of residues by specificity pocket that are predicted to interact with the phosphate and hydroxyl groups of an enantiomeric pair of docked and energy minimized inositol tetrakisphosphate substrates. Note that specificity pocket A is occupied by an hydroxyl group in a productive binding mode for hydroxy kinase activity.

pocket	Ins(1,4,5,6)P_4		Ins(3,4,5,6)P_4	
	substituent	residue	substituent	residue
A	3-OH		1-OH	
B	2-OH		6-PO_4	G295
				N272
				Y191
				R202
C	1-PO_4		5-PO_4	K20
				S23
				K298
D	6-PO_4	K20	4-PO_4	K20
		K61		K61
E	5-PO_4	K61	3-PO_4	G152
				H157
				Mg^{2+}
F	4-PO_4	G152	2-OH	
		N291		
		Mg^{2+}		

Table S3. Overview of specificity of *St*ITPK1 towards substrates and substrate analogs.

Substrate/analog	[1] Product formation (µM/µM protein)	[2] Product formation (µM/µM protein)	[3] Product formation (µM/µM protein)	[4] % inhibition at 3h
				Ins(1,2,3,4,5)P_5 substrate
Ins(3,4,5,6)P_4			326	
Ins(1,4,5,6)P_4			41	
Ins(1,2,3,5,6)P_5		0		
Ins(1,2,3,4,5)P_5		257		control
myo-InsP_6	241	289	220	89
scyllo-InsP_6	0	0		
D-*chiro*-InsP_6	0	0		
neo-InsP_6	0	0		
1-PP-InsP_5	9	75		76
3-PP-InsP_5	35	193		71
5-PP-InsP_5	0	0		82
5-PCH_2Am-InsP_5	0	25		43
5-PCF_2Am-InsP_5	0	0		67
1-PCP-InsP_5	22	145		61
3-PCP-InsP_5	41	232		78
5-PCP-InsP_5	0	0		60
1-PA-InsP_5	0	9		42
5-PA-InsP_5	50	236		44

For each column, substrate turnover values are derived from a single experiment with multiple substrates tested beside each other at 1 mM concentration. Reactions were sampled by HPLC at 3h [1] and/or after 12h [2] incubation at 25°C. [3] Results of a separate 12h experiment. [4] Extent of inhibition of Ins(1,2,3,4,5)P_5 phosphokinase activity of *St*ITPK1 by 0.5 mM competing substrate/inhibitor in a 3h experiment. Reactions that yielded products have been confirmed on at least 5 occasions. Reactions that failed to yield products (indicated by value 0) have been confirmed on at least 3 occasions.

ABBREVIATIONS

*At*ITPK1, *Arabidopsis thaliana* inositol tris/tetrakisphosphate kinase 1; *At*ITPK4, *Arabidopsis thaliana* inositol tris/tetrakisphosphate kinase 4; D-*chiro*-$InsP_6$, 1-D- *chiro*-inositol 1,2,3,4,5,6-hexakisphosphate; EDTA, ethylenediamine tetra-acetic acid; HEPES, 4-(2-hydroxyethyl)-1-piperazineethane sulfonic acid; His, histidine; HPLC, high-pressure liquid chromatography; *Eh*ITPK1, *Entamoeba histolytica* inositol tris/tetrakisphosphate kinase 1; Ins(1,4,5,6)P_4, 1D-*myo*-inositol 1,4,5,6-tetrakisphosphate; Ins(3,4,5,6)P_4, 1D-*myo*-inositol 3,4,5,6-tetrakisphosphate; Ins(1,2,3,4,5)P_5, 1D-*myo*-inositol 1,2,3,4,5-pentakisphosphate; Ins(1,2,3,5,6)P_5, 1D-*myo*-inositol 1,2,3,5,6-pentakisphosphate; Ins(1,3,4,5,6)P_5, *myo*-inositol 1,3,4,5,6-pentakisphosphate; $InsP_6$, Ins(1,2,3,4,5,6)P_6, *myo*-inositol 1,2,3,4,5,6-hexakisphosphate; 1-$InsP_7$, 1-PP-$InsP_5$, 1D-1-diphospho-*myo*-inositol 2,3,4,5,6-pentakisphosphate; 2-FAM-$InsP_5$, 2-*O*-(2-(5-fluoresceinylcarboxy)-aminoethyl)-*myo*-inositol 1,3,4,5,6-pentakisphosphate (triethylammonium salt); 3-$InsP_7$, 3-PP-$InsP_5$, 1D-3-diphospho-*myo*-inositol 1,2,4,5,6-pentakisphosphate; 5-$InsP_7$, 5-PP-$InsP_5$, 5-diphospho-*myo*-inositol 1,2,3,4,6-pentakisphosphate; $InsP_8$, [PP_2]-$InsP_4$, bis-diphospho-*myo*-inositol-tetrakisphosphate; 1,5-$InsP_8$, 1D-1,5-bis-diphospho-*myo*-inositol 2,3,4,6-tetrakisphosphate, 1,5-[PP]$_2$-$InsP_4$; 3,5-$InsP_8$, 1D-3,5-bis-diphospho-*myo*-inositol 1,2,4,6-tetrakisphosphate, 3,5-[PP]$_2$-$InsP_4$; MES, 2-(*N*-morpholino)ethanesulfonic acid; *neo*-$InsP_6$, *neo*-inositol 1,2,3,4,5,6-hexakisphosphate; NMR, Nuclear Magnetic Resonance Spectroscopy; OPLS, optimized potentials for liquid simulations; PAGE, Polyacrylamide Gel Electrophoresis; PCR, polymerase chain reaction; PDB, Protein DataBank; PEG 6000, polyethylene glycol 6000; *rac*-$InsP_8$, 1:1 mixture of 1,5-$InsP_8$ and 3,5-$InsP_8$; *scyllo*-$InsP_6$, *scyllo*-inositol 1,2,3,4,5,6-hexakisphosphate; *St*ITPK1, *Solanum tuberosum* inositol tris/tetrakisphosphate kinase 1; TLS, Translation-Libration-Screw-rotation; TLSMD, TLS, Translation-Libration-Screw Motion Determination; VIH1, *Arabidopsis thaliana* diphosphoinositol pentakisphosphate kinase 1; VIH2, *Arabidopsis thaliana* diphosphoinositol pentakisphosphate kinase 2; VSGB, Generalized Born continuum solvent model; *Zm*ITPK1, *Zea mays* inositol tris/tetrakisphosphate kinase 1; 1-PCP-$InsP_5$, *myo*-inositol 2,3,4,5,6-penta-phosphate-1-methylenediphosphonate; 3-PCP-$InsP_5$, *myo*-inositol 1,2,4,5,6-penta-

phosphate-3-methylenediphosphonate; 5-PA-InsP_5, *myo*-inositol 1,2,3,4,6-pentakisphosphate-5-phosphonoacetate; 5-PCH_2Am-InsP_5, 5-deoxy-5-(phosphonoacetamido)-*myo*-inositol 1,2,3,4,6-pentakisphosphate; 5-PCF_2Am-InsP_5, 5-deoxy-5-(phosphonodifluoroacetamido)-*myo*-inositol 1,2,3,4,6-pentakisphosphate; 5-PCP-InsP_5, *myo*-inositol 1,2,3,4,6-pentakisphosphate-5-methylenediphosphonate; 1-PCP-5-PP-Ins(2,3,4,6)P_4, 5-diphospho-*myo*-inositol 2,3,4,6-tetrakisphosphate-1-methylenediphosphonate; 3-PCP-5-PP-Ins(1,2,4,6)P_4, 5-diphospho-*myo*-inositol 1,2,4,6-tetrakisphosphate-3-methylenediphosphonate
